# Nonlinear cochlear mechanics without direct vibration-amplification feedback

**DOI:** 10.1103/physrevresearch.2.013218

**Published:** 2020-02-26

**Authors:** Alessandro Altoè, Christopher A. Shera

**Affiliations:** Auditory Research Center, Caruso Department of Otolaryngology, University of Southern California, Los Angeles, California 90033, USA; Auditory Research Center, Caruso Department of Otolaryngology, University of Southern California, Los Angeles, California 90033, USA; Department of Physics & Astronomy, University of Southern California, California 90089, USA;

## Abstract

Recent *in vivo* recordings from the mammalian cochlea indicate that although the motion of the basilar membrane appears actively amplified and nonlinear only at frequencies relatively close to the peak of the response, the internal motions of the organ of Corti display these same features over a much wider range of frequencies. These experimental findings are not easily explained by the textbook view of cochlear mechanics, in which cochlear amplification is controlled by the motion of the basilar membrane (BM) in a tight, closed-loop feedback configuration. This study shows that a simple phenomenological model of the cochlea inspired by the work of Zweig [J. Acoust. Soc. Am. **138**, 1102 (2015)] can account for recent data in mouse and gerbil. In this model, the active forces are regulated indirectly, through the effect of BM motion on the pressure field across the cochlear partition, rather than via direct coupling between active-force generation and BM vibration. The absence of strong vibration-amplification feedback in the cochlea also provides a compelling explanation for the observed intensity invariance of fine time structure in the BM response to acoustic clicks.

## I. INTRODUCTION

The peripheral auditory system transforms air-borne pressure waves into neural impulses that are interpreted by the brain as sound and speech. The cochlea of the mammalian inner ear is a snail-shaped electrohydromechanical signal amplifier, frequency analyzer, and transducer with an astounding constellation of performance characteristics. These include sensitivity to subatomic displacements with microsecond mechanical response times; wide-band operation spanning ten or more octaves in frequency; an input dynamic range corresponding to a million-million-fold change in signal energy (120 dB); and the ability to rapidly vary the response gain over 2–3 orders of magnitude while keeping the phase nearly invariant [1–3]. All of this nonlinear signal detection and analysis—attained with minimal power consumption [4,5] and little harmonic and intermodulation distortion [6,7]—is achieved not with the latest silicon technology or by exploiting the power of quantum computers, but by self-maintaining biological tissue, most of which is salty water.

**FIG. 1.**
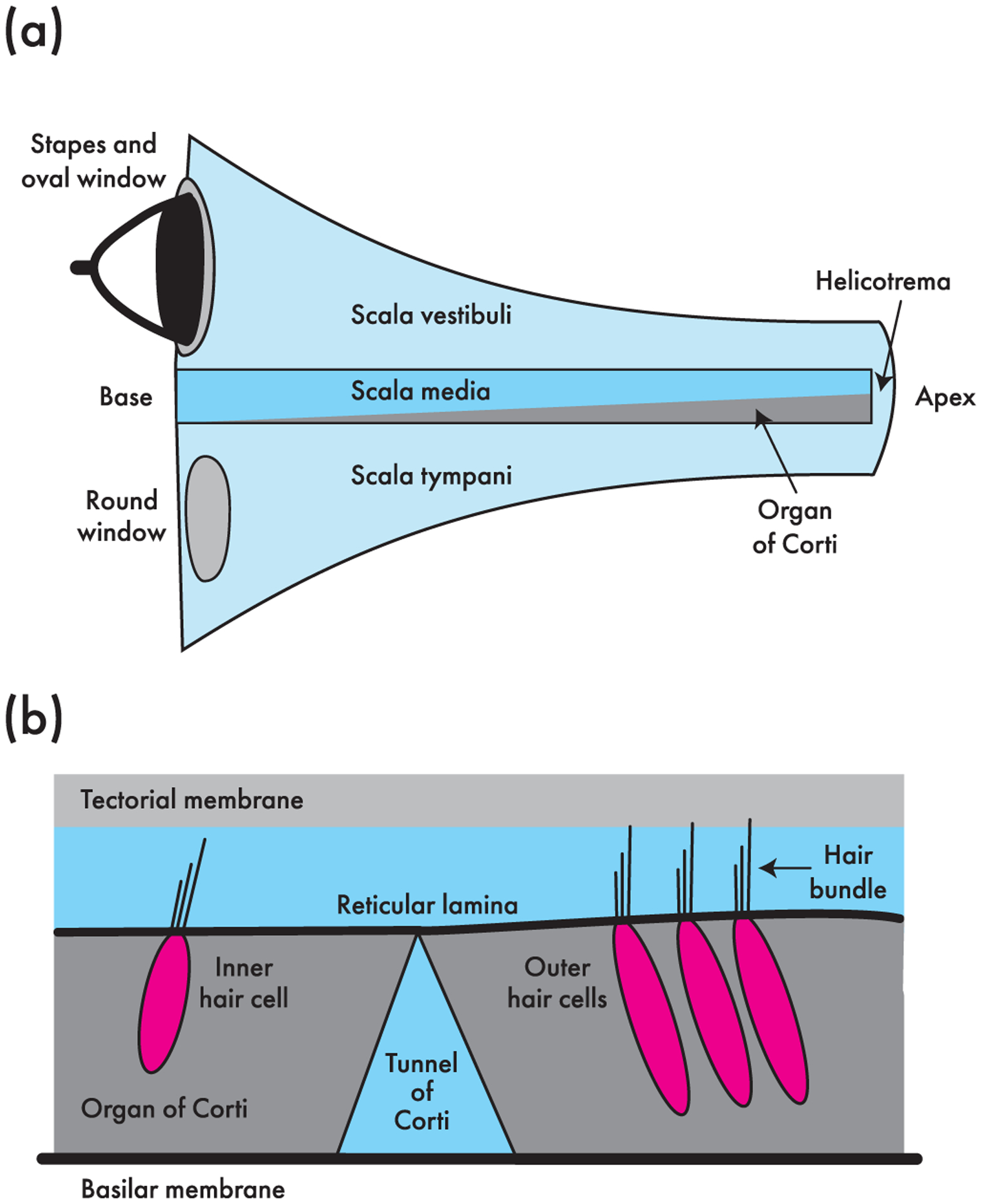
Cartoon of cochlear anatomy. (a) The cochlea consists of three fluid-filled chambers: the scala vestibuli, scala media, and scala tympani. The scalae vestibuli and tympani join at the helicotrema near the apical end of the cochlear spiral, which is shown here unrolled. The middle ear transfers sound-induced vibrations of the eardrum to the cochlear fluids via the stapes, which pushes on the oval window at the base of the cochlea. The round window, a compliant membrane in the wall of the scala tympani, separates the cochlear fluids from the air-filled middle-ear cavity and acts as a pressure release. The scala media provides a special ionic environment to the hair bundles that protrude from the reticular lamina (RL) at the upper surface of the organ of Corti. The organ of Corti (b) is the sensory organ of hearing, where mechanical vibrations are transduced by the inner hair cells (IHCs) into neural impulses that travel along the auditory nerve to the brain (not shown). In the scala media above the organ of Corti (OoC) lies the tectorial membrane, an acellular structure mechanically coupled to the organ of Corti through the stereocilia of the outer hair cells (OHCs), whose piezoelectric action helps to boost the sensitivity and dynamic range of hearing. The basilar membrane (BM), whose motion has been a principal focus of cochlear mechanics for the last century, forms the boundary between the organ of Corti and the scala tympani. Although not illustrated here, the organ of Corti comprises a variety of additional supporting cells and structures.

Understanding how the mammalian cochlea achieves its remarkable performance has been a long-standing problem in biological physics (e.g., Refs. [8–13]). Figure 1 illustrates the “spherical cowchlea”: a highly simplified representation of relevant cochlear anatomy. During normal hearing, sound-induced vibrations of the stapes launch hydromechanical waves visible in the transverse motion of the basilar membrane (BM). Waves produced by pure tones travel along the cochlea and peak at a location dependent on the sound frequency and intensity [9]. The frequency that produces the largest BM response at each location, measured at sound levels near the threshold of hearing, defines the local characteristic frequency (CF). The resulting tonotopic map (CF versus distance from the stapes) is nearly exponential [14]. Simple hydrodynamic considerations well supported by experiments [15,16] indicate that, to leading order, BM traveling waves mirror the pressure difference across the cochlear partition (organ of Corti).

Interestingly, theoretical analyses [17–20], measurements of intracochlear pressure [16], and the existence of spontaneous otoacoustic emissions [21]—narrow-band sounds generated within the cochlea and detectable using microphones placed in the external ear canal—all imply that these slow-traveling, transpartition pressure waves not only transfer energy to the organ of Corti but also receive energy from it. In other words, transpartition pressure waves appear actively amplified as they propagate to their CF place. The dominant view in the field is that cochlear amplification involves piezoelectric forces produced by the outer hair cells [OHCs, Fig. 1(b)], whose soma actively expand and contract in response to motions of the stereociliary bundle [22]. The resulting coherent wave amplification enables the cochlea to act as a biological, hydromechanical analog of a laser amplifier [20]. Although the profound contributions of this nonlinear active process to normal hearing have been appreciated for many years, there is as yet no clear consensus on how the spatially coordinated amplification is actually brought about.

Unfortunately, the daunting complexity of the cochlea, which consists of many thousands of coupled electromechanical elements that respond nonlinearly to sound, renders it impossible to deduce how the system works solely from measured data. Mathematical and computer models are therefore an essential tool for exploring the principles underlying the remarkable mechanics of the cochlea.

Both because mechanical nonlinearities appear confined to the peak region of the BM frequency response (e.g., Ref. [1]), and because inverse methods applied in either 1- or 3D consistently find a region of apparently negative BM damping basal to the peak of the BM traveling wave (e.g., Refs. [18,19]), models of cochlear mechanics almost universally posit the existence of direct mechanical feedback between BM motion and the forces generated by the cochlear amplifier (e.g., Refs. [18,23–25]). For example, 1D models often explicitly assume that the active process manifests itself on the BM as a form of nonlinear negative damping tuned in a fashion similar to that of BM motion (e.g., Refs. [18,23]).

Recently, however, the existence of a direct vibration ↻ amplification feedback loop has been challenged by mechanical measurements from the organ of Corti. Several different laboratories have now consistently observed that the mechanical responses of the outer-hair-cell (OHC) region near the reticular lamina (RL) are broadly tuned and respond to sound nonlinearly at frequencies where BM responses are nearly linear [26–30]. Furthermore, Dewey *et al*. [31] have shown that the forces that move the RL at low frequencies can be suppressed without affecting the vibration of the BM. Together, these measurements suggest that the forces underlying the cochlear amplifier are only weakly coupled to the motion of the BM. Thus the classic view of cochlear amplification based on direct vibration ↻ amplification feedback appears suspect.

Independent of these experimental developments, Zweig [32] derived a linear 3D model of cochlear mechanics based upon the pioneering recordings of Rhode from the squirrel monkey [33]. The derivation led Zweig to conclude that the BM can be represented as an array of fluid-coupled harmonic oscillators driven by the transpartition pressure and an active force proportional to the time derivative of that pressure. Interestingly, close inspection reveals that the active force in the model is broadly tuned compared to the BM. Furthermore, the force operates at low frequencies, where it only weakly amplifies the vibrations of the BM. Thus the properties of the active force in Zweig’s model bear a striking resemblance to vibrations recently measured in the OHC region of the organ of Corti.

Are these qualitative similarities between model and data mere coincidence, or has Zweig’s analysis captured something essential? Because the derivation is based on application of an inverse method to extrapolated measurements from squirrel monkeys with compromised cochlear sensitivity, there are reasons to doubt the model generality. Furthermore, the inversion procedures and their conclusions have never been tested against more modern recordings, or even in other species, most of which appear to manifest quantitatively different behavior. (For example, unlike other small laboratory mammals, squirrel monkeys have unusually long low-frequency group delays in the tail region of their transfer functions; see Sec. II.)

Starting from the simple representation of local BM mechanics deduced by Zweig [32], we fit a 3D model to BM transfer functions recently recorded in two common animal models of cochlear mechanics—the base of the gerbil cochlea and the apex of the mouse. In contrast to Zweig [34]—who concluded that the cochlear amplifier provides not only an active force but also modifies the damping and stiffness of the cochlear partition—we find that fitting the model to more recent data requires a relatively large partition damping whose value remains independent of stimulus level. This large damping, combined with the distributed effect of active forces on the BM, produces a tall and broad peak in the BM transfer function that matches experimental data from sensitive animals. Eliminating the active force produces tuning curves remarkably similar to those measured post-mortem or at very high sound levels. In addition, rendering the active force nonlinear demonstrates that the model preserves the approximate intensity invariance of fine time structure evident in BM responses to acoustic clicks (zero-crossing invariance; see Refs. [2,35]). Zero-crossing invariance is difficult to achieve in models with a mechanically tuned cochlear amplifier [36,37] and requires an intricate co-variation of parameters in more phenomenological models [2,34]. This aspect is further analyzed in Appendix A, where the BM oscillator equation derived by Zweig [32] is obtained as the simplest equation for an active model of the cochlea that respects zero-crossing invariance. Overall, the model indicates that a simple, leading-order description of organ-of-Corti mechanics, coupled with an idealized but basically realistic representation of the 3D geometry of the scalae, captures the main features of linear and nonlinear cochlear mechanics. The behavior of the active-force term also mirrors that seen in recent recordings from the organ of Corti.

## II. THE MODEL

We employ a 3D model of the cochlea based upon the work of Zweig [32] that combines the long- and short-wave approximations. Whereas Zweig focused on deriving the wavelength from the data, and therefore employed different BM admittances in the long- and short-wave regions, we embed his results about the functional form of the cochlear oscillators within a model of the cochlear fluids whose physical realization requires a geometry that is qualitatively consistent with that observed in the mammalian cochlea. The resulting model contains a simple, minimalist mechanical model of the cochlear partition. Unfortunately, because the hydrodynamics of the cochlea is rather complex, the overall model formulation is not as straightforward as the local mechanics of the partition. In particular, it has been known for a long time that the ratio between transverse and longitudinal motion of the fluids near the BM depends on both location and frequency [11,32,38,39].

Two key differences between the proposed model and that of Zweig [32,34] are the determination of model parameters and the manner of their variation in the nonlinear regime. Zweig [32] deduced the parameters of the BM impedance from mathematical extrapolations performed on Rhode’s measurements in the squirrel monkey [33]. The extrapolation to low stimulus levels shows sharp BM tuning near the characteristic frequency (CF) and unusually long group delays in the low-frequency tail region of the transfer function. Near-CF group delays are also substantially longer than those observed in other species, making them hard to reconcile with the delays of otoacoustic emissions, even in primates such as humans whose otoacoustic delays are exceptionally long [40]. The unusual sharpness and delay of the extrapolated BM transfer function lead Zweig to conclude that the effective damping of the partition must be small at low stimulus levels, where the cochlea is approximately linear.^1^ Under this hypothesis, capturing the level-dependent tuning of the BM then requires assuming that the oscillator damping increases with level, as Zweig proposed [34]. In contrast, we find that the best results are obtained by keeping the damping constant and varying only the magnitude of the active force. As detailed in Appendix A, this assumption preserves the approximate intensity-invariance of the fine time structure (e.g., the zero crossings) of the BM click response in a straightforward manner.

### A. Overview and assumptions

**FIG. 2.**
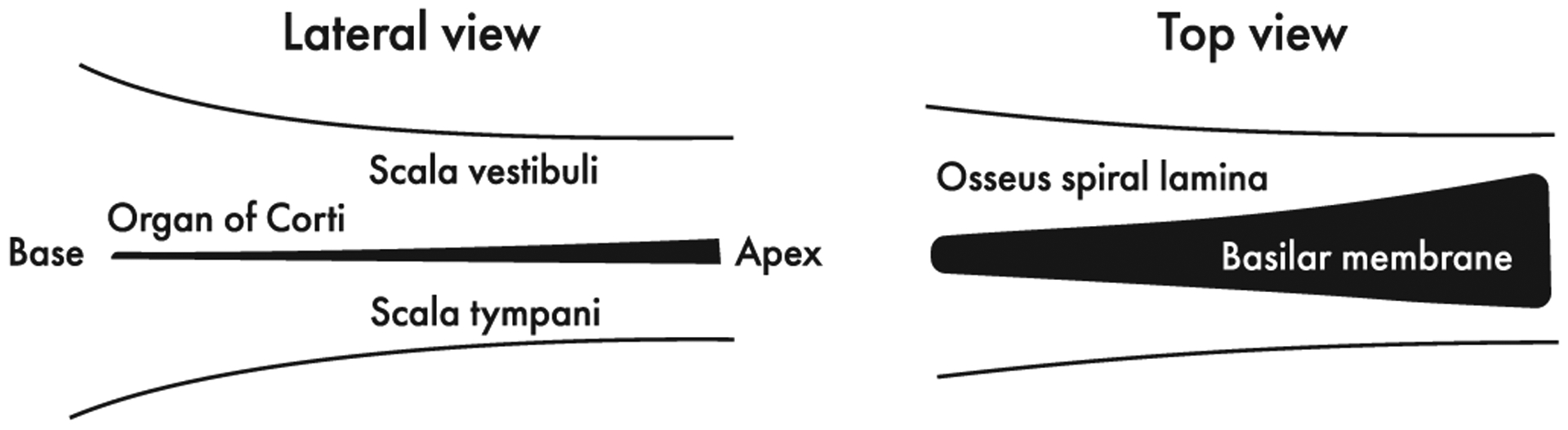
Assumed geometry of the cochlea. Whereas the scalae cross-sectional areas decrease from base to apex, the mass of the organ of Corti and the width of the BM increase. These opposing tapers are consistent with the geometry of the real cochlea and allow us to assume that the cochlear input impedance is approximately real [41]. At the same time, we assume that the tapering of scala height is gentle enough that the height can be regarded as approximately constant over the peak region of the traveling wave. This approximation greatly simplifies the equations of wave propagation in the peak region. Note that in this model the acoustic mass of the BM is determined by the mass per unit length of the organ of Corti divided by the BM width. Since the BM width is small compared to the scala width, the acoustic mass of the BM is large even though the area of the organ of Corti spans but a tiny fraction (~1%, see text) of the total cross-sectional area of the scalae.

Figure 2 illustrates the assumed geometry of the model and its opposing tapers. Whereas the effective cross-sectional area of the scala (*S*) decreases from base to apex, the area of the organ of Corti (*A*) and the width of the BM (*b*) both increase (see Secs. II C and II D). In our model, the tapering is necessary to reduce the reactive part of the cochlear input impedance (see Ref. [41]), allowing us to approximate it as real. Although the model does not incorporate a detailed anatomical description of the cochlea, its tapered geometry is consistent with morphological data (see Sec. II C). We further assume that the tapering of the scala height is gentle enough to enable Siebert’s [38] short-wave approximation in the peak region of the traveling wave (see Sec. II D).

Unlike Zweig [32], we do not assume that the motion of the BM is perfectly scaling symmetric. For tonal stimuli, scaling implies that BM transfer functions depend not on location and frequency independently but on the dimensionless ratio *ω*/*ω*_c_(*x*), where *ω*_c_/2*π* is the natural frequency of the local BM oscillator.^2^ On the other hand, we assume local scaling^3^ of the wave number of the slow-traveling pressure-difference wave that drives the motion of the cochlear partition. Thus, when the cochlear map is exponential, the pressure wave is locally shift-invariant. This assumption greatly simplifies the mathematical description of cochlear mechanics by allowing use of a single variable to express frequencies and relative distances in the cochlea (see Sec. II C).

In addition, we assume that deformations of the organ of Corti (e.g., due to internal forces) have little effect on the transpartition pressure field, and their influence (i.e., the generation of fast compressional waves) is therefore neglected.^4^ With these assumptions, the model solutions are nearly equivalent to those obtained in a 2D box model. The exception occurs in the low-frequency tail of the response, where the input impedance of the box model retains a larger reactive component.

Finally, we assume that the equations of motion obey a form of stimulus and spatial universality [34]. Thus, although the parameter values may vary along the cochlea, the equations themselves depend neither on cochlear location nor on the nature of the stimulus. Consequently, equations deduced from measurements obtained using narrow-band stimuli remain applicable when the stimulus is more complex.

### B. Basic definitions and notation

The external force per unit length acting on the BM is

(1) $$f_{\text{ext }}(x,t)=b(x)p(x,t),$$

where *p*(*x*, *t*) is the driving pressure difference across the BM and *b*(*x*) is the BM width. The effective mass per unit length of the BM is determined by the cross-sectional area, *A*(*x*), of the attached organ of Corti, defined as the effective area of the tissues and fluids that move with the BM, including the (unknown) inertial load of the TM:

(2) m(x)=ρA(x),

where we have taken the mass density of the cellular structures populating the organ of Corti to be similar to that of the surrounding fluids (*ρ*). At any location, the local mechanics of the BM are represented by a harmonic oscillator driven both by the transpartition pressure and an additional active force, *f*_act_,

(3) $$m\left({\dot{v}}_{\text{BM}}+2\zeta \omega_{\text{c}}v_{\text{BM}}+\omega_{\text{c}}^{2}\int v_{\text{BM}}dt\right)=f_{\text{ext}}+f_{\text{act}}.$$

In this equation, *v*_BM_ is the transverse BM velocity, the diacritical dot denotes differentiation with respect to time, *ζ* is the dimensionless damping coefficient, and *ω*_c_ is the natural angular frequency of the oscillator. Note that the parameters in Eq. (3) depend on *x*. Following Zweig [32], we assume that for small BM vibrations the active force is proportional to the time derivative of the external driving force (the transpartition pressure):

(4) $$f_{\text{act}}(x,t)\propto {\dot{f}}_{\text{ext}}(x,t)\propto \dot{p}(x,t).$$

where ∝ indicates proportionality. When the active process is disabled (e.g., post-mortem), *f*_act_ = 0.

To simplify the description of cochlear mechanics, we assume harmonic time dependence and adopt the complex-valued scaling variable *s*(*ω*, *x*) = *iω*/*ω*_c_(*x*), whose magnitude represents the stimulus frequency normalized by the local resonant frequency of the oscillator. As we explain below, the scaling variable *s*(*ω*, *x*) also represents relative distances in the cochlea, allowing one to describe cochlear macromechanics as a function of a single variable (*s*) instead of two (*x* and *ω*). Note that *s* = *i* when *ω* = *ω*_c_. Except in the extreme apex of the cochlea, the cochlear frequency-position map is approximately exponential [14]. Thus

(5) $$s\left(\omega_{\text{c}},x\right)=ie^{\left(x-x_{\text{c}}\right)/l},$$

where *ℓ* is the “space constant” of the map and *x*_c_ is the location whose oscillator frequency is *ω*_c_. Consequently, *ds*/*dx* = *s*/*ℓ*.

Solving for the BM admittance, defined as

(6) $$Y_{\text{BM}}(x,s)=V_{\text{BM}}(x,s)/P(x,s),$$

where *V*_BM_(*x*, *s*) and *P*(*x*, *s*) indicate BM velocity *v*_BM_(*x*, *t*) and transpartition pressure *p*(*x*, *t*) in the frequency domain, yields

(7) $$Y_{\text{BM}}(x,s)=\frac{b(x)}{m(x)\omega_{\text{c}}(x)}\frac{s(1+\tau s)}{s^{2}+2\zeta s+1},$$

where the term *τs* represents the active force [Eq. (4)]. Both the force coefficient *τ* and the damping factor *ζ* are assumed constant within the cochlear region of interest.

### C. Long-wave region

In regions far basal to the peak of the traveling wave—or, equivalently, at frequencies in the tail region of the BM frequency response—the wavelength of the traveling wave is much larger than the height of the scalae and wave propagation is well described by the long-wave model. Newton’s second law and conservation of mass imply that

(8) $$\frac{\partial P}{\partial x}=-i\omega \frac{\rho }{S(x)}U$$

and

(9) $$\frac{\partial U}{\partial x}=-b(x)V_{\text{BM}}=-b(x)Y_{\text{BM}}P,$$

where *S*(*x*) is the effective cross-sectional area of the scalae and *U* denotes fluid volume velocity.^5^ Equations (8) and (9) describe a transmission line in which the traveling, transpartition pressure wave satisfies the uncoupled equation

(10) $$\frac{d^{2}P}{dx^{2}}-\frac{d\text{ln}Z}{dx}\frac{dP}{dx}-ZY_{\text{BM}}P=0,$$

with *Z* = *iωρb*(*x*)/*S*(*x*).

Equation (10) simplifies by assuming scaling symmetry in the long-wave region; we therefore take *b*(*x*)/*A*(*x*) ~ *ω*_*c*_(*x*) and *b*(*x*)/*S*(*x*) ~ 1/*ω*_*c*_(*x*), where ~ indicates approximate proportionality.^6^ With this assumption the dependence on *x* is removed and the wave equation becomes a function of the scaling variable *s* [18],

(11) $$\frac{d^{2}P}{ds^{2}}-\kappa_{s}^{2}P=0,$$

where *κ*_*s*_ denotes the (dimensionless) complex wave number in the “*s* domain:”

(12) $$\kappa_{s}(s)=2\pi \tau_{\text{wf}}\sqrt{\frac{1+\tau s}{s^{2}+2\zeta s+1}}.$$

In this equation, *τ*_wf_ denotes the wave-front group delay expressed in cycles of CF.^7^ Under our assumptions, *τ*_wf_ amounts to a constant factor,

(13) $$\tau_{\text{wf}}=\frac{l}{2\pi }\sqrt{\frac{b^{2}(x)}{S(x)A(x)}}\approx \text{constant},$$

in agreement with the experimental data [2].

As a reality check, we used Eq. (13) to estimate the effective cross-sectional area of the organ of Corti (*A*) in the mouse by combining anatomical measurements [44,45] with estimates of the wave-front delay derived from published BM transfer functions [29]. The result yields an organ of Corti area amounting to roughly 0.4% of the total cross-sectional area of the scalae in the base and 12% in the apex.^8^ These quantities correspond respectively to equivalent partition thicknesses of 25 and 135 *μ*m in the base and apex, respectively. These effective thicknesses seem plausible, although they are perhaps somewhat too small in the base and too large in the apex. We note that the large effective acoustic masses necessary in long-wave models do not require unrealistic dimensions for the organ the Corti. Because the acoustic mass (or inertance) represents mass per unit area, the relatively large BM inertance arises from a small organ of Corti mass combined with a narrow BM.^9^

Approximate solutions to Eq. (11) for *P*(*s*) can be obtained using the WKB method [18]:

(14) $$P(s)\approx \frac{P_{0}}{\sqrt{\kappa_{s}}}\text{exp}\left[-\int_{s_{0}}^{s}\kappa_{s}\left(s^{\prime }\right)ds^{\prime }\right],$$

where *P*_0_ is the pressure applied at the base of the cochlea and *s*_0_ is the value of *s* at the base. Because |*s*_0_| ≪ 1 except at BM locations very close to the stapes, we henceforth take *s*_0_ = 0 for simplicity. When the cochlear input impedance is resistive, *P*_0_ is proportional to stapes velocity. Consequently, the long-wave BM transfer function (i.e., the ratio between either BM velocity and stapes velocity or BM displacement and stapes displacement) becomes

(15) $$T_{\text{lw}}(s)\propto \frac{Y_{\text{BM}}}{\sqrt{\kappa_{s}}}\text{exp}\left[-\int_{0}^{s}\kappa_{s}\left(s^{\prime }\right)ds^{\prime }\right].$$

### D. Short-wave region

The traveling wave slows down dramatically as it approaches the peak region, where the wavelength becomes short enough that the long-wave approximation no longer applies. Indeed, the wavelength becomes much smaller than the height of the scalae [32,38,49], and the short-wave approximation, in which the scalae heights are assumed effectively infinite, is well suited to describe the propagation of the traveling pressure wave. To employ the short-wave approximation, we must also assume that the tapering of the cochlea occurs on a scale long compared to the local wavelength, so that the geometric parameters can be regarded as essentially constant in the peak region. With this simplifying assumption, we can use Siebert’s equation for a wave traveling from the base to the apex [38]:

(16) $$\frac{1}{2\rho \omega }\frac{dP}{dx}=Y_{\text{BM}}(x,\omega )P(x,\omega ).$$

Using Eq. (7) and noting that *ω* = *ω*_c_|*s*| yields

(17) $$\frac{dP}{dx}=\frac{2i\rho b}{m}\frac{s\left|s\right|(1+\tau s)}{s^{2}+2\zeta s+1}P,$$

leading to

(18) $$\frac{dP}{ds}-\kappa_{s}P=0,$$

where the short-wave wave number is

(19) $$\kappa_{s}=2\pi \tau_{\text{sw}}\frac{\left|s\right|(1+\tau s)}{s^{2}+2\zeta s+1}.$$

Our assumptions imply that the parameter

(20) $$\tau_{\text{sw}}=bl/(A\pi )$$

is independent of *x* in the short-wave region.

Zweig [32] argues that the short-wave approximation becomes invalid past the peak of the traveling wave, where the finite scalae heights play a role in determining the peculiar shape of the BM transfer function above CF (e.g., sharp notches accompanied by sudden phase jumps). We further note that the short-wave equation breaks down near *s* = *i*. In particular, the real part of the wavelength changes sign at a frequency above CF whose exact value depends on the model parameters. The unphysical consequence is that the short-wave solution for the forward-traveling wave reverses itself and becomes a backward-traveling wave.^10^ For these reasons, we show model results only for |*s*| < 1, where the solution remains valid.

### E. Joining the two regions

Combining results for the long- and short-wave regions gives

(21) $$\kappa_{s}=\{\begin{array}{ll} 2\pi \tau_{\text{wf}}\sqrt{\frac{1+\tau s}{s^{2}+2\zeta s+1}}, & \left|s\right|⩽\left|s_{\text{t}}\right|\\ 2\pi \tau_{\text{sw}}\frac{\left|s\right|(1+\tau s)}{s^{2}+2\zeta s+1}, & \left|s_{\text{t}}\right|<\left|s\right|<1 \end{array},$$

where *s*_t_ denotes the “transition point” beyond which the long-wave approximation fails and the solution is assumed short-wave. Unlike the situation in the real cochlea, where the transition between the long- and short-wave regions occurs smoothly, albeit presumably over a relatively small spatial region, this “chimeric” approach introduces a discontinuity in the complex wave number across *s*_t_. A reasonable approach to joining the solutions in the two regions is to require that the (real) wavelength remains continuous at the transition. This constraint,

(22) $$\underset{s\to s_{t}^{+}}{\text{lim}}\text{Re}\left[\kappa_{s}\right]=\underset{s\to s_{i}^{-}}{\text{lim}}\text{Re}\left[\kappa_{s}\right],$$

suffices to determine the parameter *τ*_sw_. Once the wave numbers are everywhere determined, the BM velocity (and consequently the BM transfer function) can be computed to within a constant factor:

(23) $$V_{\text{BM}}(s)\propto \{\begin{array}{ll} \frac{P_{0}Y_{\text{BM}}}{\sqrt{\kappa_{\text{s}}}}\text{exp}\left[-\int_{0}^{s}\kappa_{s}ds^{\prime }\right], & \left|s\right|⩽\left|s_{\text{t}}\right|\\ P_{\text{t}}Y_{\text{BM}}\text{exp}\left[-\int_{s_{\text{t}}}^{s}\kappa_{s}ds^{\prime }\right], & \left|s_{\text{t}}\right|<\left|s\right|<1 \end{array},$$

where *P*_t_ is the pressure at the transition point.

### F. Reality check in a box model

To verify that the shotgun wedding we performed above—the coerced conjunction of long- and short-wave solutions—leads to no unphysical progeny, we compare the results of the calculations against those performed in a 2D box model of the gerbil cochlea. In particular, we use the equation of the BM admittance [Eq. (7)] in a 2D box model [49,51]. The parameters of the 2D box model are the same as those of the 3D model. The height of the scalae in the 2D model is 0.65 mm (scala tympani and scala vestibuli areas of 0.4225 mm^2^), based on gerbilline values from reference [52]. As discussed in Sec. II A, we expect the 2D box model to produce solutions close to those of the chimeric model except in the low-frequency tail of the response, where the input impedance of the box model retains a larger reactive component than the chimeric model.

### G. Nonlinearities

We model the cochlear nonlinearity by assuming that the only nonlinear element resides in the active-force term. In particular, we assume that the linear active force *f*_act_ ∝ *τ ṗ* represents the first term of an expansion of a nonlinear function of *ṗ*. Hence, the nonlinearity can be modeled by making *τ* a nonlinear function of *ṗ*. Implementing this ansatz in the normalized-frequency (*s*) domain,^11^ we take

(24) $$\tau (s)=\tau_{0}(1-\text{tanh}\left(\left|sP(s)\right|/{\dot{P}}_{\text{sat }}\right),$$

where *τ*_0_ is the value of *τ* in the linear regime (determined by fitting transfer functions measured at low sound levels), *Ṗ*_sat_ is a constant that controls the activation of the nonlinearity, and *sP* represents the time derivative of the pressure in the frequency domain.

Assuming that harmonic distortions produced by responses to pure-tone stimuli have a negligible effect on the action at the fundamental frequency, we adopt an iterative scheme to compute the nonlinear responses via Eq. (23). Our scheme is similar to that previously used by Kanis and de Boer [53]. In particular, at each iteration step, we compute the solution numerically over the spatial range of interest (i.e., the appropriate range of *s*) and then update the value of *τ* at each location according to Eq. (24). We stop the iteration and deem the solution convergent when two successive solutions differ by less than some criterion (in our case, 1%). This method is conceptually similar to the method of averaging (e.g., Ref. [54]), where the nonlinearity is assumed to depend on the response envelope. Because the envelope of the response to a steady tone is constant over time, we need only iteratively estimate the traveling-wave envelope to obtain an approximate solution adequate to account for the activation of the nonlinearity. This method computes nonlinear effects on steady-state responses and does not require making assumptions about the kinetics of the nonlinearity.^12^

### H. Zero-crossing invariance

The fine time structure (e.g., zero crossings) of BM and auditory-nerve responses to acoustic clicks is nearly independent of stimulus intensity (see Ref. [2]). This approximate symmetry implies invariance of both the wave-front delay and the characteristic oscillation frequency of the BM. In the model, both the conjugated pole of the BM admittance—and therefore the BM oscillation frequency—and the low-frequency group delay are independent of the nonlinear term. Consequently, the two conditions above are approximately satisfied when *τ* < 1/|*s*_t_|.^13^ Appendix A provides a heuristic derivation of the model equations based on zero-crossing invariance.

### I. Experimental data

We compare model predictions with experimental data collected in two common animal models of mammalian hearing, gerbil and mouse. The gerbil data, from the Dong laboratory at Loma Linda University, consist of BM responses to pure tones made with laser Doppler vibrometry. The mouse data, from the Oghalai laboratory at the University of Southern California, were collected with optical coherence tomography (OCT) using the methods outlined in Ref. [31]. Both gerbil and mouse recordings provide BM velocity and ear-canal pressure (i.e., the sound pressure measured near the stimulus loudspeaker). Because the gerbil middle-ear transfer function is approximately constant [55], we assume a simple proportionality between ear-canal pressure and stapes velocity in this species. Thus we approximate the gerbil BM transfer function (BM motion re stapes) by the ratio of BM velocity to ear-canal pressure. In contrast to the gerbil, the mouse middle-ear transfer function resembles a first-order high-pass filter (6 dB/octave) in the frequency range analyzed here (1–15 kHz) [56]. We therefore assume a proportionality between ear-canal pressure and stapes displacement and approximate the BM transfer function by the ratio of BM displacement to ear-canal pressure. In both mouse and gerbil, we correct phase responses for the middle-ear delay, which amounts to approximately 20 *μ*s in both species [56]. Although the delay between loudspeaker and tympanic membrane remains unknown, adding an additional delay of approximately 5–10 *μ*s yields slightly different parameter values but has no qualitative effect on our conclusions.

## III. RESULTS

### A. Gerbil model

#### 1. Responses at low sound levels and post-mortem

The parameters of the model can be adjusted to fit BM transfer functions measured in different species; the values for gerbil and mouse are reported in Table I. In this section, we focus on the responses of the model tailored to recordings from the base of the gerbil cochlea (CF ~ 17–21 kHz). We compare the predictions of the 3D chimeric and 2D box models with BM transfer functions measured under conditions where the cochlea behaves almost linearly: *in vivo* at low sound levels and post-mortem. Because *in vivo* responses evoked by low-level tones at frequencies in the low-frequency tail region are too small to be measured, we compare model responses in this region, where BM responses are approximately linear, with data obtained at slightly higher levels.

The 3D model requires the determination of four parameters; the box model only three. First, we set the parameter *τ*_wf_ by extrapolation from the group delay of the experimental data in the low-frequency tail of the response (*τ*_wf_ = 0.95). Second, in the chimeric model we need to define the transition frequency between the long- and short-wave regions (|*s*_*t*_|); this transition is an emergent property of the 2D box model. Based both on simulations of the 2D model and on the location of the change of slope in the magnitude and phase of the measured data, we selected |*s*_*t*_| = 0.435, which corresponds to a frequency approximately 48% of CF. Finally, the parameters *τ*_0_ and *ζ* can be empirically determined by noting (i) that *ζ* controls the transfer function post-mortem, when *τ*_0_ = 0; (ii) that *τ*_0_ controls the gain of the transfer function *in vivo* relative to post-mortem; and (iii) that both *τ*_0_ and *ζ*_0_ affect the bandwidth of the peak. From these observations, we found that *ζ* = 0.15 and *τ*_0_ = 1.25 produce satisfying fits to the data.

**FIG. 3.**
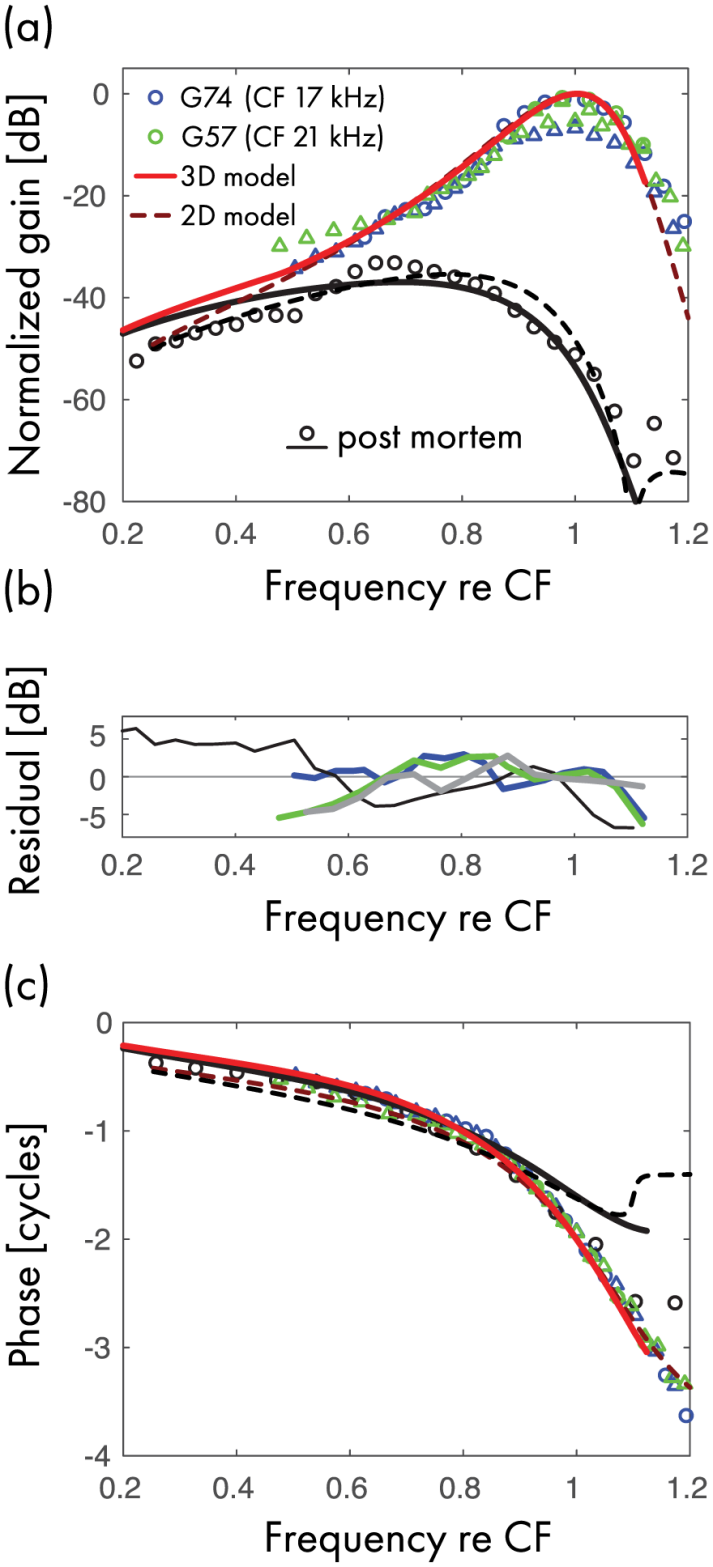
Comparison between measured and model BM transfer functions in gerbil. [(a) and (c)] The model *in vivo* and post-mortem transfer functions are shown in red and black, respectively. The unconnected symbols represent measured transfer functions from the base of two gerbils (CF indicated in legend). The green symbols represent measurements from animal G57 at 30 (◯) and 50 dB (Δ) SPL; the blue symbols represent data from animal G74 at 30 (◯) and 40 dB (Δ) SPL. The solid lines show the results calculated in the 3D “chimeric” model, while the dashed line are results from the 2D box. Because the model predicts BM transfer functions only to within an overall complex constant, we normalize the transfer function by the peak magnitude measured at the lowest SPL *in vivo*. (b) Gain difference between the predictions of the 3D model and the experimental data. The blue and green lines give the difference between the model prediction and the experimental data from animals G74 and G57, respectively. The black line shows the difference with the post-mortem data, and the gray line that between the two animals.

Figure 3 compares the linear BM transfer functions predicted by the model with those measured *in vivo* and post mortem. The solid and dashed lines show results from the 3D “chimeric” and the nonscaling symmetric 2D box models, respectively. Small differences between the responses of the two models are evident at low frequencies, where the models have different values for the cochlear input impedance. Computing the transfer function of the 2D model as the ratio between the BM velocity and the transpartition pressure at the base of the cochlea (rather than the stapes velocity) reduces the discrepancy between the 2D and 3D transfer functions to a couple of dB (not shown).

Both models predict similar transfer-function magnitudes and appear consistent with the experimental data. The 2D box model, whose numerical solution is accurate at all frequencies, predicts a plateau in the amplitude and phase of the post-mortem BM transfer function beyond the peak, in agreement with the data. The “*in vivo*” 2D box model also predicts a notch followed by a plateau above the frequency range displayed in Fig. 3.^14^

Figure 3(b) plots the residual (difference in dB) between the gain of the transfer function predicted by the model and the experimental data. For comparison, Fig. 3(b) also shows the difference between the measurements from the two gerbils. Over the range of frequencies analyzed, model predictions differ by less than 5.5 dB from the experimental data, a range similar to the intersubject variability manifest in the data. Consistent differences between the *in vivo* data and the model responses can be seen at frequencies about 20% below CF, where the magnitude data show a downward inflection not captured by the model. Also, at about 10% above CF the model predicts a steeper magnitude cutoff than is evident in the *in vivo* data. Both models fit the *in vivo* phase profile quite well. A discrepancy between the model and the data is apparent in the somewhat larger phase difference between the *in vivo* and post-mortem conditions at high frequencies.

Such discrepancies presumably reflect one or more of our simplifying assumptions. These includes adopting an uncomplicated form for the active force, neglecting possible short-range effects of cochlear tapering when solving the model equations, ignoring viscoelastic longitudinal coupling between cochlear elements, and disregarding the internal structure and deformation of the organ of Corti. Although other parameter choices allow the model to better capture specific features of the measured BM transfer function,^15^ the model plainly has too few adjustable parameters to fit all details seen in the data. Nonetheless, the model captures the major trends, and the differences appear small in light of the simplicity of the model mechanics.

#### 2. Responses in the nonlinear regime

**FIG. 4.**
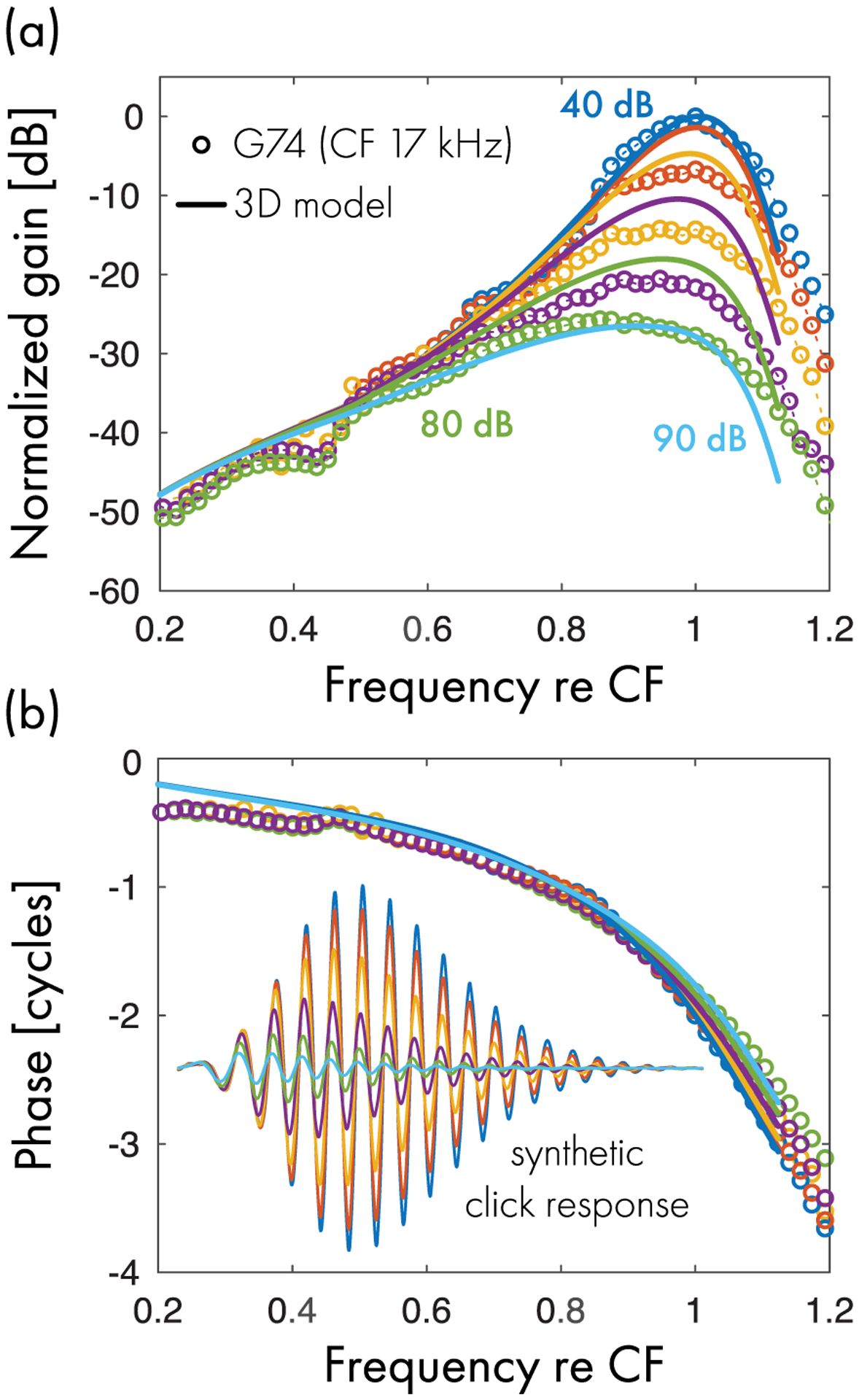
Comparison between model and measured BM transfer functions in the base of the gerbil cochlea (CF = 17 kHz) at various stimulus levels (10 dB steps). (a) Peak-normalized gain and (b) phase. The solid line shows the predictions of the 3D chimeric model; the open symbols show the experimental data. Different colors encode different stimulus levels. The inset in (b) shows the model synthetic click responses obtained by inverse Fourier transforming the transfer functions.

Nonlinear responses to tones can be simulated in the chimeric model by using an iterative approach to estimate the envelope of the traveling wave on the BM (see Sec. II F). Figure 4 compares model BM frequency responses with experimental data from gerbil obtained at different stimulus levels. Although the model does not match the data precisely, the predicted magnitudes are in good qualitative agreement with the measurements. The phase responses match the data very well, showing only subtle variations with level below CF. We note, however, that although the model captures the overall shape and level dependence of the response magnitude, the model requires a larger variation of stimulus intensities to match the full dynamic range evident in the data. Although adopting a different nonlinear function in Eq. (24) might allow the model to better capture the level dependence of the BM response, fine-tuning the nonlinearity is beyond the scope of the present work, for which we opted to maintain simplicity in the model equations.

Close inspection of Fig. 4 reveals another limitation of the model, which fails to capture the downward inflections evident in the magnitude data at frequencies corresponding to approximately 40% and 80% of CF. These inflections are accompanied by wobbles in the phase; at frequencies near 0.4CF the phase slope even becomes briefly positive (negative group delay). Roughly speaking, the data resemble the result of adding a small, quasiperiodic oscillation to a curve that otherwise closely approximates the predictions of the model. These observations suggest that the measured response contains a small standing-wave component, in addition to the dominant forward-traveling wave. Standing waves can arise through multiple internal reflection [58] via processes that are not represented in the model, which lacks both micromechanical irregularity to scatter the traveling wave and a reflective boundary condition at the stapes.

Without a time-domain implementation of the nonlinear model, we currently have no precise way of investigating the level dependence of the model response to acoustic clicks. Approximate methods include reducing the active force along the entire cochlea by a constant factor or computing synthetic click responses by inverse Fourier transforming the BM frequency response. Naively, the first method might appear justified by de Boer’s EQ-NL theorem [59], but the theorem applies only for continuous broadband stimuli such as noise. The second method suffers from the problem that the spatial pattern and time course of nonlinear compression along the cochlea differs between clicks and tones. Notwithstanding these technical caveats, the two strategies produce similar results, both experimentally and in the model. For example, inverse transforming BM frequency responses measured with tones produces synthetic click responses that closely match those measured with clicks, except in the late ringing portion of the response [35], which is often contaminated by internal reflection. The inset in Fig. 4(b) illustrates the intensity dependence of the model synthetic click responses. As expected, the model exhibits approximate zero-crossing invariance, which reflects the phase invariance of the BM transfer function (see Sec. II H and Appendix A).^16^

### B. Mouse model

**FIG. 5.**
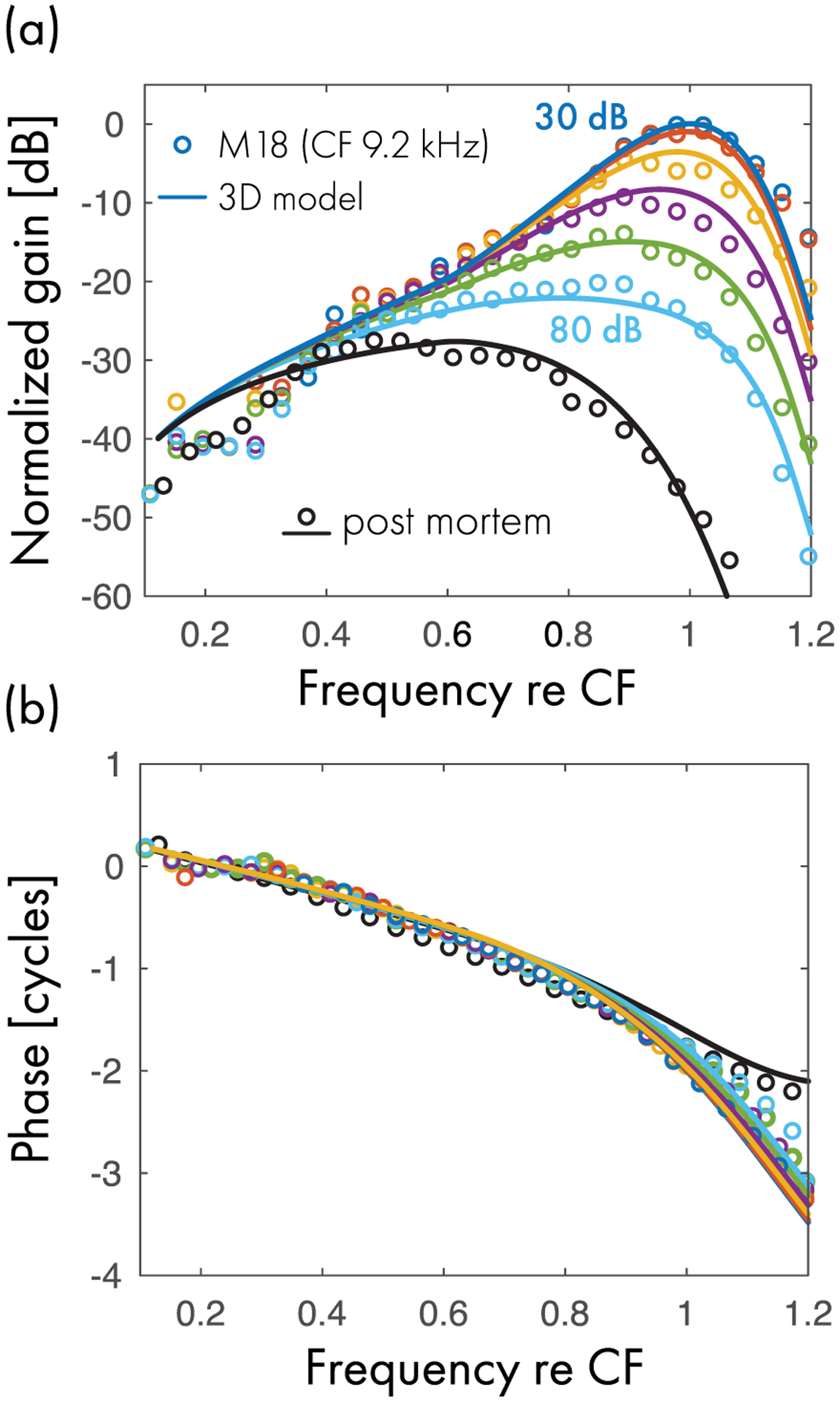
Comparison between model and measured BM transfer functions in the apex of the mouse cochlea (CF = 9.2 kHz) at various stimulus levels (30–80 dB SPL in 10 dB steps). (a) Peak-normalized gain and (b) phase. The connected symbols represent the experimental data. The solid lines show the predictions of the 3D chimeric model. Different colors encode different stimulus levels. The black lines and symbols represent post-mortem responses.

Figure 5 shows the model fits to BM measurements from the apex of the mouse cochlea. Parameter values are given in Table I. The model provides a good fit to both the magnitude and phase of the data at all levels tested. As with the gerbil data, the model misses a downward magnitude inflection, and corresponding region of negative group delay, apparent in the tail region of the response (in this case, around two octaves below CF). Once again, the inflection may indicate the presence of a standing-wave component in the response due to internal reflections not present in the simple model.

## IV. DISCUSSION

### A. Modeling approaches

Cochlear models can be broadly categorized into two partially overlapping classes. The first consists of models built around detailed representations of cochlear micromechanics and its material properties (e.g., Refs. [39,60,61]). These models provide useful tools for exploring how the various anatomical substructures within the organ of Corti contribute to shaping response features seen experimentally (e.g., Refs. [61–65]). Although such models can successfully reproduce existing data, their complexity—albeit still considerably less than that of the real cochlea—remains high. Indeed, they generally have many degrees of freedom and a corresponding number of uncertain parameters. Consequently, models in this class run the risk of missing the forest for the trees.

Models in the second class arise from a complementary perspective that averages over the trees to focus on the forest. They achieve insights into cochlear mechanics not from the bottom up, but from the top down; that is, by providing approximate functional relationships between measurable variables, such as those between the pressure across the partition and the motion of the BM (i.e., the BM admittance). In conjunction with a simplified macroscopic account of wave propagation in the cochlear fluids, these models employ lumped-element representations of the mechanics of the partition; by comparison with the models in the first class, they are coarse-grained and perhaps slightly out of focus.

Depending on the model, the representations in this second class can be either deduced from data as solutions to appropriate “inverse problems” (e.g., Refs. [18,66]), hypothesized from considerations of structural and functional anatomy (e.g., Refs. [23,24,67]), or imposed as universal descriptions imported from dynamical systems theory (e.g., Refs. [68–70]). Although they usefully exploit approximate symmetries in the data, the resulting equations seldom retain an unambiguous mechanical interpretation and they therefore provide only a phenomenological account of cochlear micromechanics. Compared to their more detailed brethren, these models provide less overtly realistic but considerably more tractable representations of the cochlea. Ideally, they are both simple enough to help identify general mechanisms that underlie specific patterns in data (e.g., Refs. [25,41,71]) and specific enough to make testable predictions about how different phenomena correlate with one another (e.g., Refs. [72,73]). Their computational efficiency lends them practical utility for simulating cochlear responses to complex signals such as speech, and they can therefore be used for understanding the perception of sound, including the role of the cochlea’s nonlinear signal processing in shaping physiological responses obtained from other stages of the auditory system [36,74,75]. Despite differences in their levels of explanation, models in the two classes are mutually informative and must, one presumes, ultimately converge on a consistent description of cochlear mechanics.^17^

### B. Consistency with the physics of the real cochlea

Our focus here has been on a model in the second class. Conceptually, the model is very simple. (1) The organ of Corti is represented by an array of harmonic oscillators that move in proportion to the local displacement of the BM. (2) The BM inertance is made consistent with the width of the BM and the mass of the attached organ of Corti. (3) The local damping parameter is assumed constant, independent of BM motion. (4) The motion of the BM is presumed driven by the transpartition pressure and an additional force that boosts the mechanical response, rendering the model active and nonlinear. (5) The active force is approximated as perhaps the simplest that fulfills the conditions necessary to achieve intensity invariance of the zero crossings of BM click responses (see Appendix A).

The model suggests that the tall, broad peak of the *in vivo* traveling wave is produced by three interacting factors: (i) a passive BM resonance at a frequency lower than the natural frequency; (ii) a build-up of propagated driving pressure caused by the active-force term; and (iii) the transition between long- and short-wave behavior that further enhances the driving pressure near the peak of the traveling wave (see, e.g., Ref. [49]).

The most interesting feature of the model—and also its greatest enigma—is the active-force term, which depends on the time derivative of the pressure [32]. Although presumably a proxy for forces created by outer hair cells, the active force offers no immediate micromechanical interpretation. In the following section, we explore the implications of this peculiar active force and show that its behavior is consistent with recent measurements of cochlear micromechanics.

### C. The active force resembles relative BM and RL motion

Since the discovery of OHC electromotility [22], the idea that traveling-wave amplification involves negative or antidamping—that is, an active force operating roughly in-phase with BM velocity near the best place [18,23,24]—has dominated thinking in the field. A common corollary is that the active forces are tuned to provide maximal local amplification near the characteristic frequency. Both theoretical (e.g., Refs. [36,76]) and experimental studies (e.g., Ref. [77]) have therefore sought mechanisms capable of generating the narrowly tuned forces thought responsible for the sharp frequency tuning manifest in basilar-membrane motion.

**FIG. 6.**
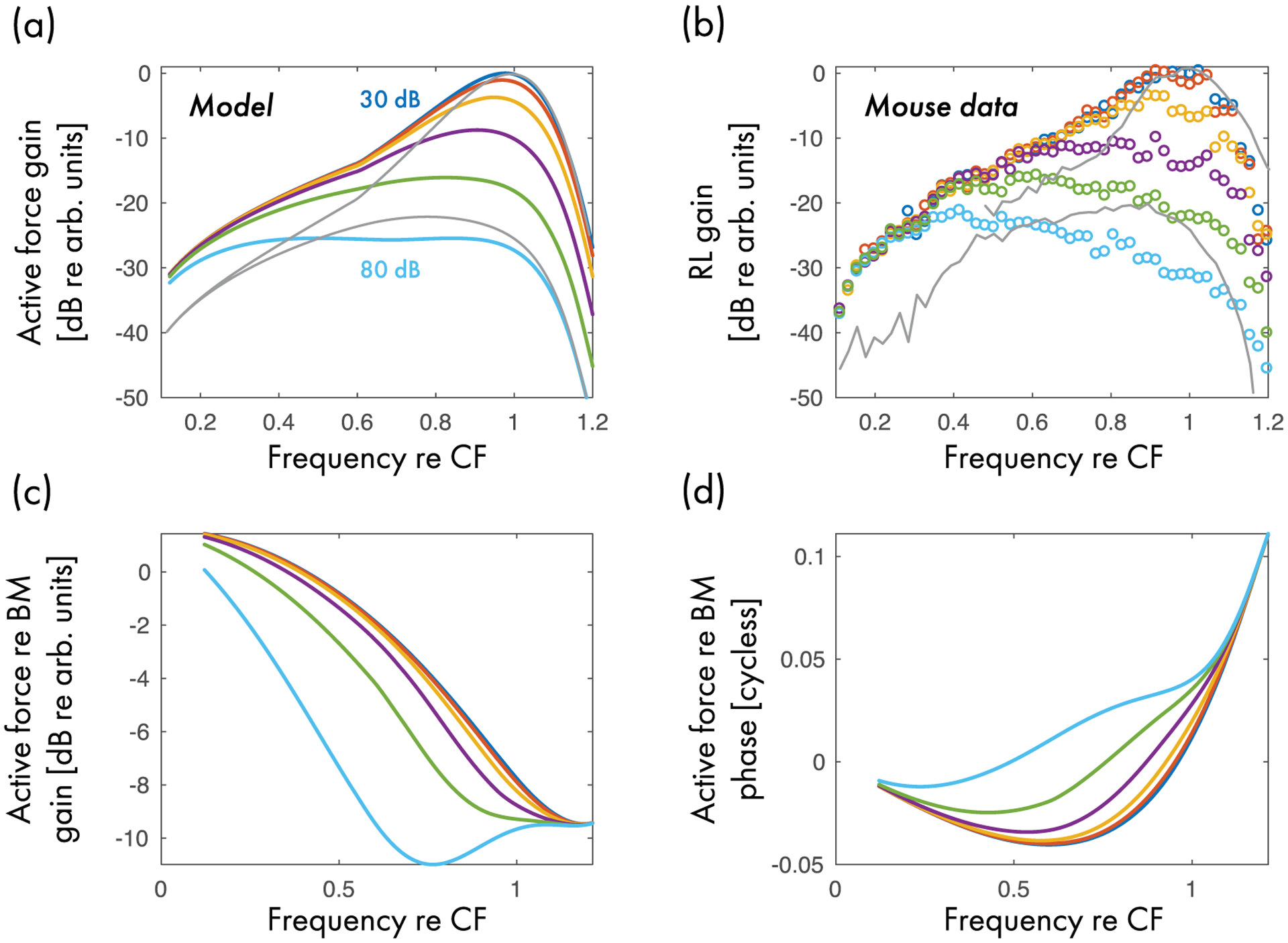
(a) Gain of the active force relative to the pressure at the base of the cochlea (*sτP*/*P*_0_) in the mouse model at different stimulus levels (10 dB steps). (b) Vibration gain (relative to ear-canal pressure) of the OHC region (“RL”) measured in the same mouse whose BM responses are shown in Fig. 5. The thin gray lines in (a,b) show the BM gain of Fig. 5 at the lowest and highest sound levels tested. (c) Ratio of active-force gain to BM velocity gain. (d) Phase of the active force relative to that of BM velocity.

Following Zweig [32]—who obtained the result by reinterpreting his one-dimensional inverse solution in three dimensions—the present model assumes instead that the active force driving the BM depends not directly on local BM velocity or displacement but on the time derivative of the driving pressure. The consequences of this assumption are markedly different from the classic view of a vibration ↻ amplification feedback loop in the cochlea. As demonstrated in Fig. 6(a), which shows the magnitude of the active force in the mouse model at different levels of stimulation, the active force is broadly tuned (relative to BM motion) and nonlinear even at tail frequencies, where BM responses are linear. [For reference, the thin gray lines in Figs. 6(a) and 6(b) replot the BM gain of Fig. 5(a) at the lowest and higher sound level tested.] Figure 6(c) plots the ratio of the active-force gain to BM velocity gain, demonstrating that the relative magnitude of the active force reaches a minimum near the BM response peak.^18^ Thus the principal contribution made by the active forces to BM tuning and amplification occurs not via a direct “push-pull” action on the BM. Rather, the sharpening of BM tuning occurs as the active forces “tune” the driving pressure as the traveling wave propagates towards its best place (see Sec. IV E). The cochlear amplifier manifests itself as a broadband force that provides narrow-band amplification of BM motion.

Ultimately, the value of these observations depends on identifying plausible connections between the mechanisms at play in the model and those operating in the cochlea. Unfortunately, relating the behavior of the model active force to relevant aspects of cochlear micromechanics—most obviously, perhaps, to the relative motions of the BM and RL—faces considerable uncertainty, both theoretical and experimental. For example, the relative motions of the BM and RL are influenced by multiple, mutually entangled factors, including the mechanical impedances of these two structures; the internal OHC forces acting upon them, either directly or via the TM; and the external forces that control the motion of their center of mass. Furthermore, phase shifts arising both from the traveling wave and from complex three-dimensional movements of the organ of Corti render the experimental measurement of micromechanical motions—including the relative phases of BM and RL vibration—extremely sensitive to the orientation of the laser beam used for the recordings [30].

Despite these interpretive uncertainties—none of which are unique to the present report—some features of the model can be qualitatively related to existing data. Assuming, for example, that the active force in the model represents the action of OHC somatic motility, the action-reaction principle suggests that the OHC force acting on the RL can be equated with the model active force, but pointing in the opposite direction. Under this assumption, the tuning and extent of nonlinearity of the active-force term appears consistent with the nonlinear responses evident in measurements of RL vibrations at low frequencies [Fig. 6(a)], where BM responses are linear [26,27,29,30]. For comparison with the model active force shown in Fig. 6(a), panel (b) shows the magnitude of RL vibration (normalized to ear-canal pressure) measured concurrently with the BM data used as the reference for determining the model parameters. The similarity between the active force and the measured vibrations of the RL, both in terms of their frequency tuning and their level dependence, is striking [Figs. 6(a) and 6(b)].

Although the model contains no mechanical representation of the RL, and can therefore make no explicit prediction about its motion, the unconventional active term seems to provide a compelling phenomenological description of OHC-generated forces. Indeed, the relationship between BM motion and active-force magnitude appears qualitatively similar to that observed between BM motion and extracellular potential—an electrical correlate of OHC force—in the gerbil cochlea [16]. Certainly, the active force in the model appears more consistent with recent experimental findings than a force tuned as or more sharply than BM velocity (e.g., Refs. [18,36]).

Figure 6(d) plots the phase of the active force relative to BM velocity. The force appears nearly in phase with BM velocity below CF and then transitions to approach the phase of BM acceleration at higher frequencies. Again, under the assumption that the active force in the model represents OHC generated forces, application of the action-reaction principle implies that the force pushes and pulls on the RL in a direction opposite to BM motion up to CF and in the same direction well above CF. Further, because the gain of the active force decreases relative to that of BM motion as the wave approaches CF [Fig. 6(c)], the model suggests that the motion produced by external forces (e.g., the transpartition pressure, which accelerates the center of mass of the organ of Corti) becomes progressively more important relative to the motion produced by internal, OHC-generated forces in determining the overall motion of the RL near CF. These model suggestions seem compatible with the studies by Ren and colleagues [26,27,78] that provide evidence for (i) antiphasic and larger RL than BM motion at low frequencies and (ii) similar BM and RL motion (magnitude and phase) approaching CF. By contrast, amplification by means of sharply tuned OHC forces (i.e., amplification dominated by the local contraction and elongation of the OHCs) would be expected to introduce the largest differences between RL and BM motion near CF.

### D. Wavelengths and the long- and short-wave approximations

**FIG. 7.**
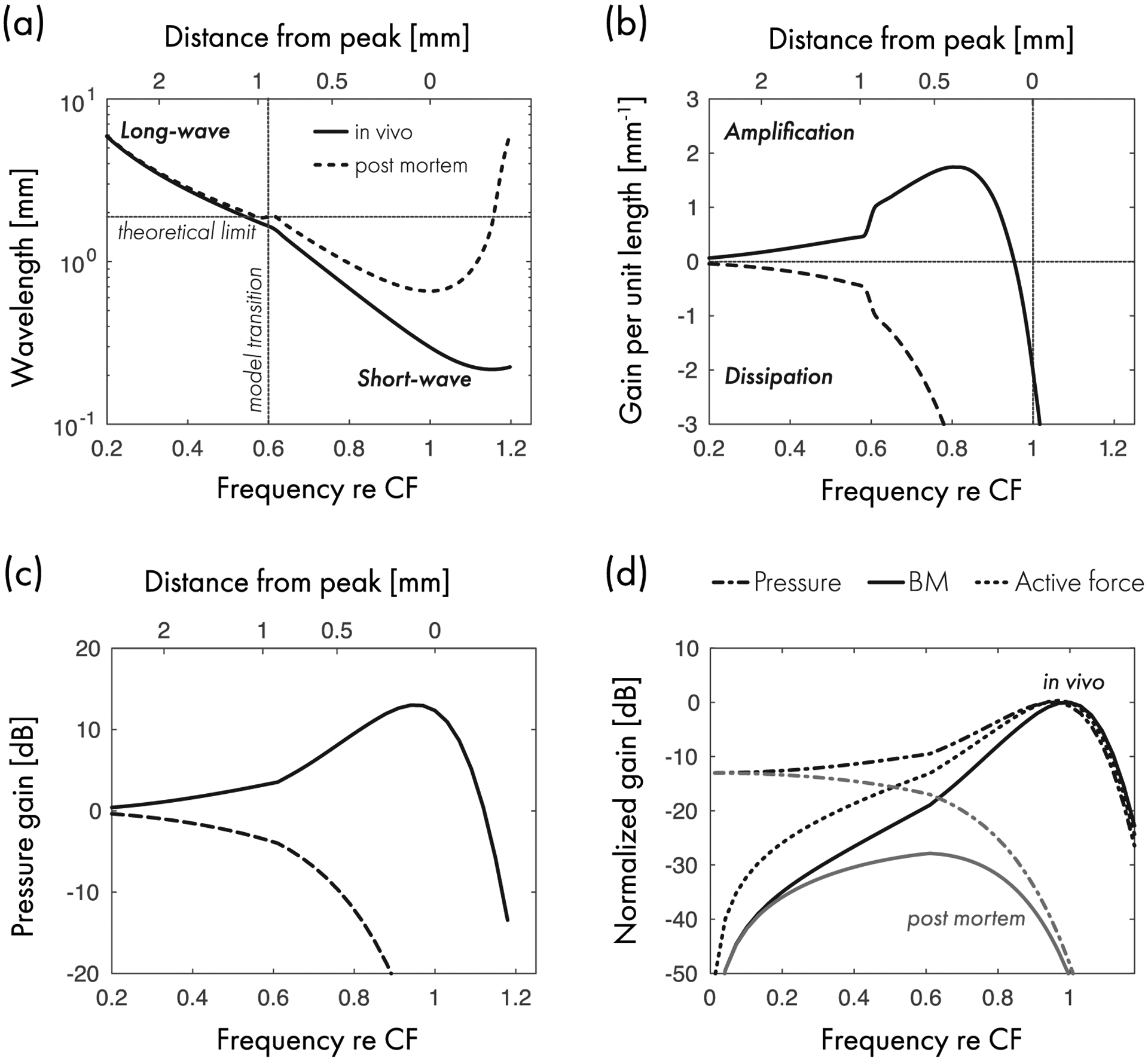
(a) Real part of the wavelength of the pressure wave in the mouse model. The dashed horizontal line bisects the plane into an upper region where the long-wave approximation is valid, and a lower region where the approximation breaks down (scalae height *H* ≈ 0.3 mm). The dashed vertical line marks the transition between long and short-wave regions in the model. (b) Gain per unit length (imaginary part of the wave number). The vertical line marks the place of maximal BM velocity for the *in vivo* model. Note that the peak of the pressure wave is determined by the zero crossing of the gain function. (c) Gain of the driving pressure relative to the pressure at the base of the cochlea. The space constant of the mouse cochlear map was taken as *ℓ* ≈ 1.8 mm [46]. (d) Comparison of peak-normalized model gain functions for the pressure, active force (relative to pressure at the stapes), and BM motion (relative to stapes motion). Note that the active force is abolished post mortem.

Figure 7(a) plots the wavelength (real part) of the traveling pressure wave for the mouse model as a function of frequency relative to CF. Solid lines give the wavelength in the low-level linear regime; dashed lines give the wavelength post mortem. Under the assumption of local scaling, which enables relative frequency to be re-expressed as relative spatial location, the top axis on the figure indicates the corresponding distance from the peak of the traveling wave evoked by a tone at CF. Both the *in vivo* and post-mortem wavelengths decrease as the wave approaches its best place, meaning that the wave slows down in the peak region. Both wavelengths also begin to increase again beyond the peak. (Note that the peak of the traveling wave shifts basally post mortem.)

By taking the height of the scalae in the mouse apex as *H* ≈ 0.3 mm [44], one can bisect the plane into two regions (horizontal dotted line): an upper region, where the long-wave approximation is theoretically valid (*λ* > 2*πH*), and a lower region where the approximation breaks down. The vertical dotted line marks the location of the model transition between assumed long- and short-wave behavior, where the solutions have been forcibly conjoined.^19^ Although proximity of inter-section was not a criterion for selecting the transition point, the wavelength curves cross the theoretical limit of validity close to the vertical line. In the region just apical to the vertical line, neither the long- nor the short-wave conditions are fully satisfied. Thus the chimeric approach employed here extends both the long- and short-wave solutions slightly beyond their ostensible regions of validity. Fortunately, the existence of this “zone of mutual invalidity” creates few problems—in the transition region the responses of the chimeric model are almost identical to those obtained in the computational 2D box model (see Fig. 3).

### E. The spatial build-up of amplification

Experiments that interfere with normal OHC function—for example, by selectively damaging the cochlea [79] or by reducing prestin-based somatic motility via photoinactivation [80]—have demonstrated that the amplification of BM traveling waves occurs over a relatively short region basal to the peak. Using OCT and a two-tone suppression paradigm to extend these experiments to structures beyond the BM, Dewey *et al.* [31] recently explored the amplification and spatial build-up of both BM and RL traveling waves in the apex of the mouse cochlea. For tones presented at the CF of the measurement site, they found that the amplification of both BM and RL vibrations is spatially distributed, extending basally from the best place over a distance of ~1 mm. However, for tones below CF, where only the motion of the RL appears amplified and nonlinear, amplification is spatially restricted (i.e., local). In a nutshell, the data suggest that although the OHCs produce significant forces over a large region basal to the peak of the traveling wave, the effects of these forces vary systematically with location. Far basal to the peak, the forces produce local amplification of the RL, but not of the BM; near the wave peak, however, the amplification “builds-up” over space, consistent with the idea that the active process boosts the propagating pressure wave, leading to simultaneous amplification of RL and BM motion.

The presence in the model of a nonlinear force operating at low frequencies but not amplifying the vibration of the BM has been discussed in Sec. IV C. The simplest way to determine whether the model predicts a spatial build-up of amplification consistent with the data is to analyze the complex wave number (*κ*) of the traveling pressure wave. In particular, the imaginary part of the wave number describes how the gain changes over space. Regions with Im *κ* > 0 provide net power gain to the traveling wave, regions with Im *κ* < 0 provide net dissipation, and regions with Im *κ* = 0 are lossless. The cumulative traveling-wave gain (*G*) over the interval [0, *x*] is given in terms of Im *κ* by

(25) $$G(x)\approx \text{exp}\left[\int_{0}^{x}\text{Im}\kappa \left(x^{\prime }\right)dx^{\prime }\right].$$

We refer to Im *κ* as the “gain function” [20]; it represents the log-gain per unit length.

Figure 7(b) shows the gain function predicted by the model for both *in vivo* and post-mortem conditions. *In vivo*, the model predicts net amplification (Im *κ* > 0) at all frequencies below CF—and, equivalently, at all locations basal to the peak. When the model transitions from the long- to the short-wave region, Im *κ* has a small discontinuity at the seam. The gain function approaches zero at low frequencies and becomes negligible at distances more than 2 mm basal to the peak. The positive gain function corresponds to a net power amplification of approximately 10 dB.^20^ The largest contributions to the power gain occur in a region spanning about 1 mm just basal to the peak, in excellent agreement with experiment [31].

Post mortem, the gain function is always negative, as required for a passive system. In contrast to the gain functions in other models, which are positive only within a small region immediately basal to the peak of the BM traveling wave [18,19,23], the gain function in the present model is positive everywhere basal to the best place. Well below CF, however, the gain function is small enough that it has negligible effects on the model response. Overall, the gain function in the model corresponds well with conclusions about power amplification in the gerbil cochlea drawn by Dong and Olson [16]. At the lowest sound levels, they found a region of negative resistance extending about one octave below CF; at lower frequencies, the BM admittance appeared largely stiffness dominated.

The downward zero crossing of the *in vivo* gain function determines the location where the transpartition pressure wave peaks. In the model, the pressure wave peaks slightly basal to the peak of the wave on the BM, as is evident in Fig. 7(d), which compares the transfer function gain for pressure and BM motion. The two peaks need not be coincident because the tuning of BM velocity reflects the product of the pressure and the BM admittance. Recall that the model nonlinearity depends not directly on the BM velocity but on the tuning of the pressure. This, combined with the observation that the pressure wave peaks basal to the BM best place, renders the model consistent with experimental observations showing that the strongest compression and suppression of BM motion measured at the CF place is elicited by tones at frequencies slightly higher than CF (e.g., Refs. [31,81]).

The difference between the *in vivo* and post-mortem gain functions determines the dynamic range of the cochlear nonlinearity manifest in the pressure. Figure 7(c) depicts the pressure gain (i.e., the pressure relative to its value at the base of the cochlea) for the *in vivo* and post-mortem models. The gain difference is less than 2 dB at frequencies more than 1.5 octave below CF and becomes negligible more than two octaves below CF. Thus, although the model is, strictly speaking, active and nonlinear at all frequencies, the effects of the active term on both the pressure and the BM well below CF are small enough [Fig. 7(d), see also Figs. 4 and 5] that their responses appear essentially linear in the low-frequency tail region of the response.

In summary, the model represents the cochlear amplifier as an active nonlinear force operating along the entire cochlea. Nevertheless, the active force produces significant nonlinearities in the motion of the BM only in a narrow spatial region basal to the peak of the traveling wave. The model thus meshes nicely with the picture of cochlear mechanics emerging from recent measurements [26–29,31].

### F. Physical interpretation of the active force

When interpreted as a local force generated by the OHCs, the active force term in the model appears in qualitative agreement with recent experimental findings. However, the biophysical mechanisms producing this phenomenological force—a force proportional to the time derivative of the driving pressure—remain obscure. Here, we examine the force term from several different angles in hopes of providing a possible physical interpretation of its peculiar form.

First, we note that the model requires only that the force transfer function relating transpartition pressure (*P*) and active force *F*_act_ be

(26) $$\frac{F_{\text{act}}}{P}\sim s,$$

where the symbol ~ here stands for “approximately proportional to.” Equation (26) need not hold in the cut-off region beyond the peak, where *P* → for |*s*| ≫ 1. Roughly, but conservatively, we therefore assume that the proportionality applies for |*s*| ⩽ 1. With this limitation, Eq. (26) states that *the active force is approximately a first-order high-pass filtered version of pressure*.

Under the assumption that OHC somatic motility is the main source of cochlear amplification, it is theoretically possible to decompose the force transfer function into its main physiological components. For example, the transfer function [Eq. (26)] can be regarded as the result of the mechanical displacement of the OHC stereocilia, which controls the mechanoelectrical transduction (MET) current, which drives the OHC receptor potential, which in turn controls the force produced by the OHCs. Unfortunately, there is currently little consensus about the precise functioning of any of these mechanisms. So, although one could design a detailed physical model that implements the necessary phenomenology, we take to heart an admonition attributed to John von Neumann: “There’s no sense in being precise when you don’t even know what you’re talking about.” Without reviewing the many controversial details surrounding the mechanisms that regulate somatic motility, we conclude more or less where we began—a physical interpretation of the model active force in terms of OHC forces requires that the combination of all processes results in a force approximately proportional to a high-pass filtered version of the driving pressure, whose approximate tuning is depicted in Fig. 6(a). How, or whether, this approximate proportionality is achieved in the cochlea remains to be seen.

**FIG. 8.**
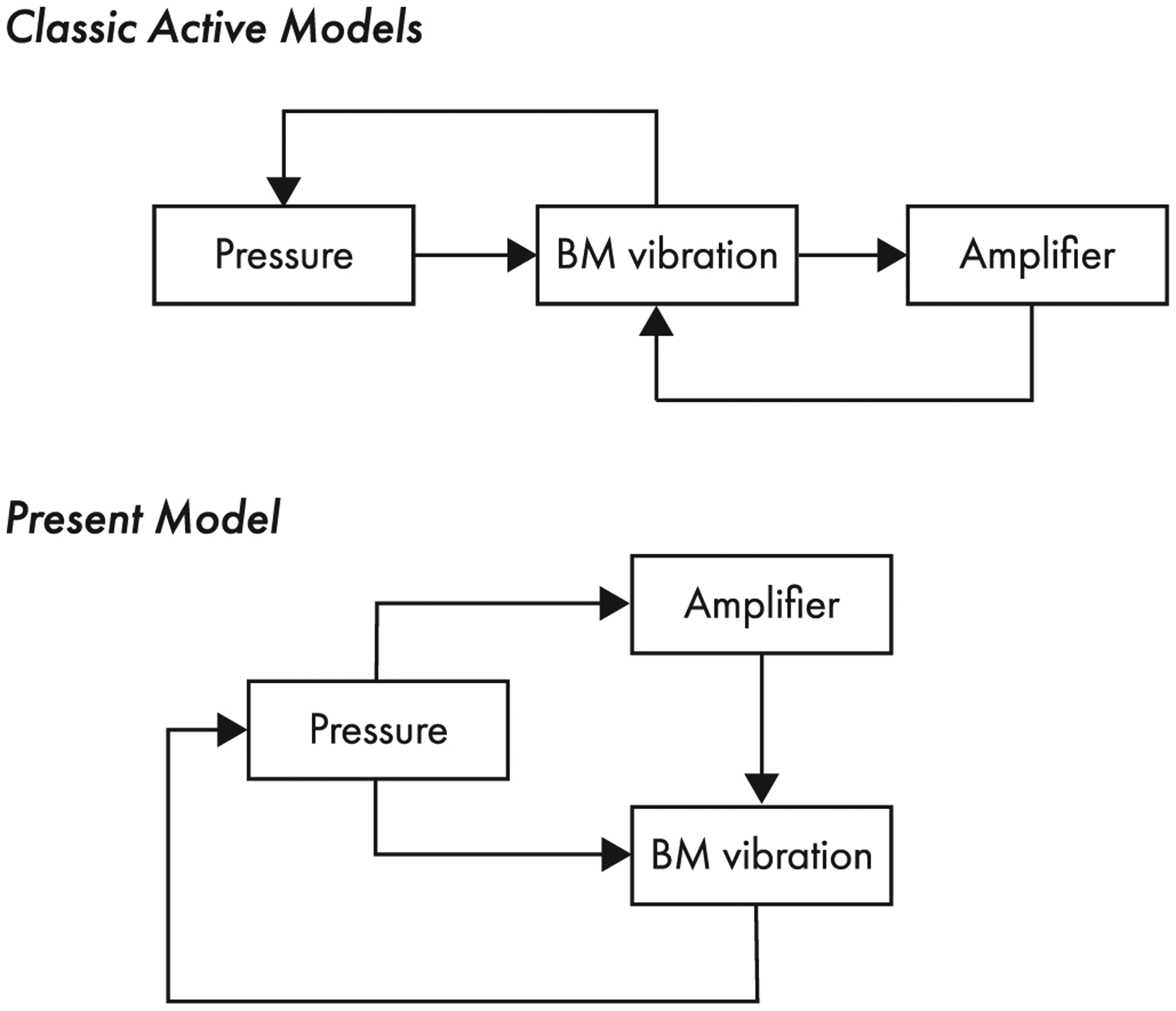
Schematic diagrams depicting causal relationships in classic active models of cochlear mechanics (e.g., Refs. [18,23,24]) and in the present model. In classic models, the transpartition pressure drives the motion of the BM, which drives the cochlear amplifier. The cochlear amplifier drives the vibrations of the BM, whose motion affects the transpartition pressure. Classic models thus employ one “passive” feedback loop to relate BM motion and pressure, and one active feedback loop to represent the action of the cochlear amplifier. Although details of the feedback system relating the cochlear amplifier and BM motion vary from model to model, the causal relationships depicted in the figure are the same in most active models. In the present model, the transpartition pressure drives the BM and the cochlear amplifier at the same time. Thus, in contrast to classic models, the present model includes only a single feedback loop, which relates pressure and BM motion.

We note, however, that although the model active force provides only an approximate phenomenological description of the biophysical processes operating in the real cochlea, the close correspondence between model predictions and recent experimental observations is possible only because the model active force is regulated fairly independently of the vibration of the BM. Feedback between the motion of the BM and the strength of the active force occurs only indirectly, via the effect of organ-of-Corti transverse vibrations on the transpartition pressure field. Figure 8 compares and contrasts illustrative diagrams depicting causal relationships in classic models and in the present model. Whereas classic models employ, in effect, two feedback loops—one from BM motion to transpartition pressure and one from the cochlear amplifier to BM motion—the present model employs only one: from BM motion to transpartition pressure. Hence, the classic vibration ↻ amplification feedback loop has been “cut” in the present model.

## V. CONCLUSIONS

Cutting direct feedback between BM motion and active force allows the present model to incorporate active forces that are nonlinear at all frequencies but that render BM vibrations nonlinear only in the peak region of the traveling wave. In classic models, the cochlear amplifier acts, in effect, to boost local mechanical resonances in the partition. As a consequence, classic models are often extremely sensitive to small variations in their parameters, or rely on oscillators that hover on the verge of instability. By contrast, the amplifier in the present model acts by enhancing the near-CF hydrodynamics, and is therefore locally stable and robust; in that sense, the model is perhaps more biologically realistic. Reducing the feedback also provides a simple and compelling interpretation of the zero-crossing invariance of BM click responses (see Appendix A). For these reasons, we suggest that the dominant view of cochlear amplification as a tight, sharply tuned closed-loop feedback system (see e.g., Refs. [82–84]) appears incompatible with the experimental data. To remain qualitatively consistent with recent experiments, it appears necessary to eliminate—or at least seriously relax—any direct feedback between BM motion and the production of active forces. Removing vibration ↻ amplification feedback from the axioms of cochlear mechanics calls into question representations of the organ of Corti by means of coupled nonlinear oscillators operating near a critical point of instability (or bifurcation, e.g., Refs. [68,70,85]).

From a mechanical perspective, cutting vibration ↻ amplification feedback requires mechanisms in the cochlea that effectively decouple the transverse motion of the BM from the vibrations of the hair bundle that drive OHC somatic motility. These mechanisms could take several nonexclusive forms. For example, compliant supporting cells may decouple the vibration of the BM from that of the top of organ of Corti, via mechanisms analogous to those of passive suspension systems in automobiles. In this regard, we note that Nobili and Mammano [86] proposed that the OHCs are coupled to the BM via compliant supporting cells, so that the cochlear amplifier operates primarily through viscous rather than elastic forces. This theory—which has been confirmed *ex vivo* by Scherer and Gummer [87,88]—can be readily probed *in vivo*, for example by comparing the tonic (DC) contraction of the OHCs with the baseline position shift of the BM [89].

Another obvious possibility is that radial shearing motions between the RL and the tectorial membrane (TM), into which the tips of OHC hair bundles are inserted, are the principal drivers of somatic motility, rendering activation of the active process only indirectly dependent on the transverse motion of the organ of Corti. In this regard, we note that previous modeling studies suggest that motions of the TM and those of the BM can indeed be fairly independent [67,90].

Because a variety of linearized models can reproduce the main features of measured BM transfer functions in the low-level linear regime (e.g., Refs. [18,23,24,65,91]), evaluating models based on their linear responses is a necessary but insufficient measure of “quality.” As Zweig pointed out [32], different linear models, even those with different numbers of spatial dimensions, can be considered functionally equivalent if they predict similar wave numbers for the traveling wave. However, when linearly equivalent models are made nonlinear, they may well behave differently, allowing them to be distinguished and tested against experiment. This work originated in simple considerations about cochlear nonlinearity and model dimensionality and their possible implications for BM phase invariance (see Appendices A and B). Unexpectedly, the arguments lead to a depiction of the active cochlea that differs substantially from the common view (see Fig. 8).

Whether and how the detailed biophysical mechanisms operating in the cochlea combine to yield an effective, collective action well captured by the phenomenological description underlying our model remains an important empirical and theoretical question. No matter how that story may ultimately play out, the model reproduces key linear and nonlinear responses of the cochlea and it can therefore be employed to the benefit of engineering and other applications requiring *in silico* models of the cochlea (e.g., Refs. [92–96]).

## Figures and Tables

**FIG. 9.**
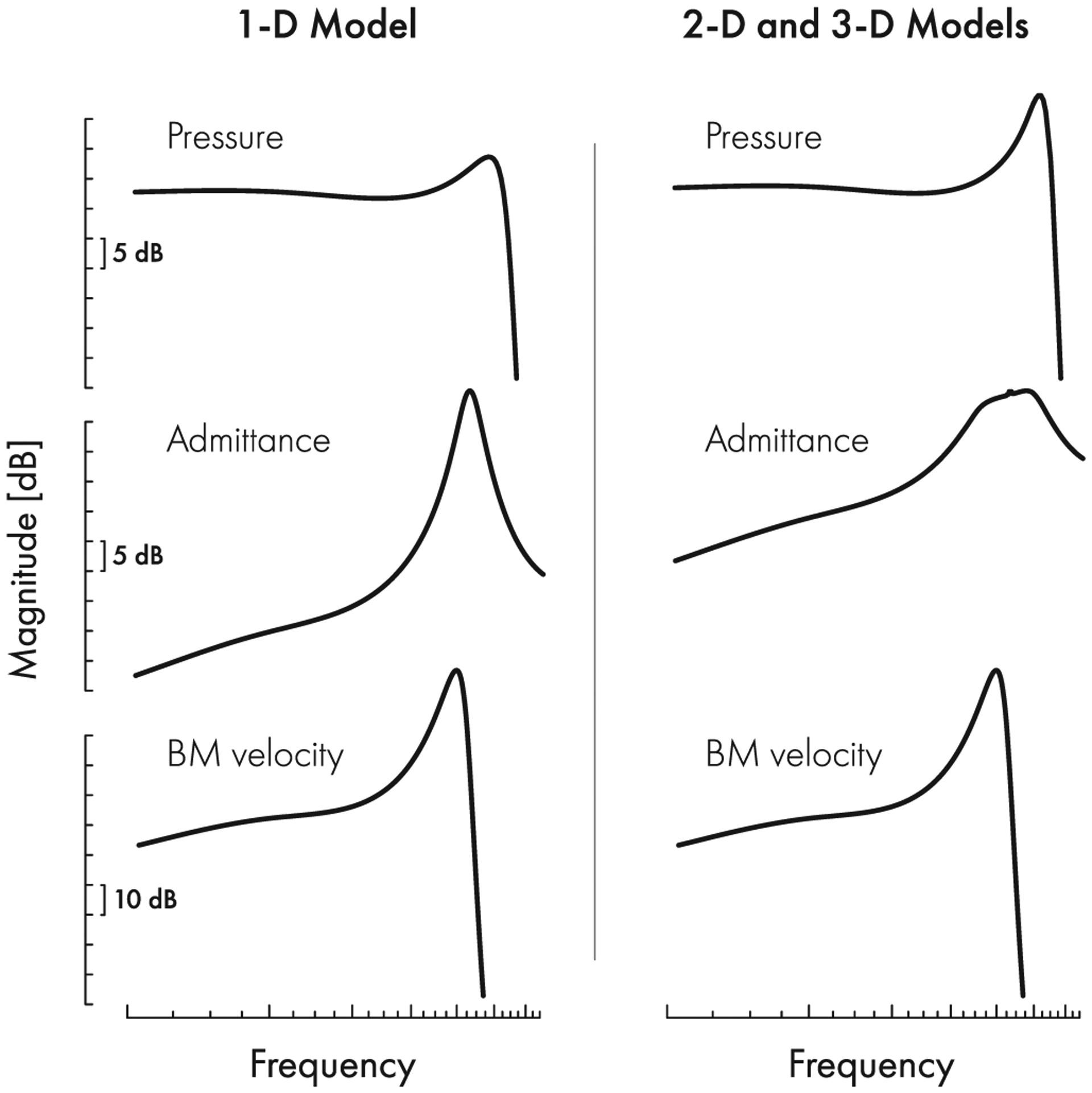
Comparison between the frequency response magnitudes of Zweig’s 1D time-delay model [18] and those of equivalent 2D or 3D models that produce the same BM frequency response.

**TABLE I.** Parameter values for the models tailored to gerbil and mouse.

Parameter	Gerbil	Mouse
τ_wf_	0.95	1.8
|*S*_*t*_|	0.435	0.5
*ζ*	0.15	0.22
τ_0_	1.25	1.2
*ℓ* (mm)	2.1	1.8

## Footnotes

1 In his solution to the inverse problem, Zweig found an oscillator with negative damping stabilized by a delayed feedback force [32]. In this case, the effective dimensionless damping can be obtained by taking the real part of locations of the principal poles of the BM admittance. The resulting value (~0.03) is much much less than one.

2 Note that the natural frequency of the model oscillators (*ω*_c_/2*π*) differs from the characteristic frequency (CF) of the transfer function. Whereas the natural frequency is an internal parameter of the model, the CF is an emergent property defined as the best frequency of the BM velocity response at low stimulus levels.

3 Perfect scaling is assumed only within the peak region of the BM traveling wave; see Sec. II D.

4 This assumption, which implies that the center of mass of the organ of Corti moves approximately proportionally to the BM, requires further experimental validation. As justification for adopting it here, we note that whereas deformations of the top of the organ of Corti (i.e., at the level of the RL) are nonlinear at frequencies for which the BM responses are linear [26–30,42], intracochlear pressure measurements show that the pressure near the organ of Corti is nonlinear only at frequencies where BM responses are nonlinear [15].

5 Denoting the areas of scala tympani and scala vestibuli by *S*_*t*_ and *S*_*v*_, respectively, then *S* = *S*_*t*_∥*S*_*v*_ = *S*_*t*_*S*_*v*_/(*S*_*t*_ + *S*_*v*_). The variable *U* represents the fluid volume-velocity difference between the two scalae.

6 The assumption of approximate scaling symmetry in the long-wave region is equivalent to the assumptions that (1) the cochlear input impedance is approximately real [43] and (2) the onset-delay of the BM click response, expressed in cycles of CF, is approximately constant in the base of the cochlea [2]. Condition (1) requires that $Z(x)m(x)\sim \omega_{\text{c}}^{-2}$ [41], while (2) requires *Z*(*x*)/*m*(*x*) ≈ constant [see Eq. (13)].

7 The quantity 2*πτ*_wf_ is denoted 4*N* by Zweig [18], where *N* represents the number of wavelengths of the traveling wave along the BM. We changed the notation for two reasons. First, *τ*_wf_ is a delay that can be directly estimated from the slope of the phase of the BM transfer function at low frequencies. Second, the relationship between wave-front delay and the number of wavelengths of the traveling wave along the BM is expected to differ in 1D and 3D models.

8 For simplicity, we assumed that scala vestibuli and scala tympani have equal areas [0.22 mm^2^ in the base (CF ≈ 50 kHz) and 0.075 mm^2^ in the apex (CF ≈ 10 kHz)] [44]. We took the BM width to be 0.07 mm and 0.135 mm in the base and apex, respectively [44,45]. We estimated the wave-front delay by fitting a line to the low-frequency phase of the mouse BM transfer function published in Ref. [29], leading to *τ*_wf_ ≈ 1.5 cycles of CF = 10 kHz (0.15 ms group delay). The space constant of the cochlear map is taken to be *ℓ* = 1.8 mm [46].

9 The effective mass of the BM in the real cochlea is determined by the combination of the inertia of the fluids (external forces acting on the organ of Corti dependent upon ${\dot{v}}_{\text{BM}}$) and by the attached mass of the organ of Corti and the tectorial membrane load. Depending on the particular assumptions and perspectives adopted by modelers, different terms are used to represent the effective inertia (mass) of the cochlear partition, which may or may not account for the inertia of the surrounding fluids (see, e.g., Refs. [39,47,48]). Our model defines the organ of Corti as a boundary between the two scalae, driven by transpartition pressure. Hence, our mass term does not include the inertial terms representing forces transmitted by the surrounding fluids. These forces are accounted for in the long and short-wave approximation equations for pressure, which are both derived from Newton’s second law for an irrotational, incompressible, and inviscid fluid (see [38]).

10 In the post-mortem case (*τ* = 0), the short-wave wavelength approaches zero in the limit |*s*| → 1, meaning that the wave slows down indefinitely and never travels beyond that point. This general phenomenon is known as an *acoustic black hole* [50]. While the slow-down of the traveling-wave phase velocity may play a role in suppressing cochlear reflections (i.e., there may be a quasi-black-hole effect), both 3D models (e.g., [32]) and experimental data showing phase and amplitude plateaus above CF, indicate that the black hole predicted by short-wave models is an artifact of the breakdown of the short-wave approximation past the peak of the traveling wave.

11 At any given location *x* we measure time in local, normalized units (periods of the local natural resonant frequency) and define *t*_*x*_ = 2*πt*/*ω*_c_(*x*). Taking *p*(*x*, *t*_*x*_) = |*P*| sin(|*s*|*t*_*x*_ + *φ*) gives *ṗ*(*x*, *t*_*x*_) = |*sP*| cos(|*s*|*t*_*x*_ + *φ*). The linear active force $\left(f_{\text{act}}^{\text{L}}\right)$ therefore has the form $f_{\text{act }}^{\text{L}}\propto \tau_{0}\dot{p}=\tau_{0}\left|sP\right|\text{cos}\left(\left|s\right|t_{x}+\varphi \right)$. If *g*(·) is the nonlinear function, the nonlinear force is $f_{\text{act}}^{\text{NL}}\propto g\left(\tau_{0}\dot{p}\right)$. In the *s* domain, the corresponding components of the active force at the fundamental frequency are $F_{\text{act}}^{\text{L}}(s)=F_{x}\left\{f_{\text{act}}^{\text{L}}\left(t_{x}\right)\right\}$ and $F_{\text{act}}^{\text{NL}}(s)=F_{x}\left\{f_{\text{act}}^{\text{NL}}\left(t_{x}\right)\right\}$, respectively, where $F_{x}\left\{\cdot \right\}$ is the Fourier transform with respect to local, normalized time. Note that $F_{\text{act}}^{\text{L}}(s)\propto \tau_{0}\left|sP(s)\right|$. Neglecting harmonic distortions, we can therefore write the nonlinear force in the form

$$F_{\text{act}}^{\text{NL}}(s)\sim \tau_{0}\bar{g}(s)\left|sP(s)\right|,$$

where $\bar{g}(s)=F_{\text{act}}^{\text{NL}}(s)/F_{\text{act}}^{\text{L}}(s)$. When the nonlinear function *g*(·) is compressive, *ḡ*(*s*) decreases when |*sP*(*s*)| increases. Not knowing the precise form of *g*, we assume that *τ*_0_*ḡ*(*s*) can be approximated by Eq. (24).

12 Harmonic distortions do not greatly affect the response at the fundamental frequency in models with instantaneous nonlinearity (see Ref. [53]).

13 In the long-wave region, the zero of the BM admittance becomes a pole of the pressure in Eq. (14). Hence, if the location of this zero (1/*τ*) occurs within the long-wave region and changes with level, the resulting pressure acquires a level-dependent phase, producing a large violation of zero-crossing invariance. Conversely, if the zero is far from the long-wave region, its variation does not greatly affect the phase of the pressure.

14 Notches and plateaus above CF are often attributed to the interaction between transpartition (slow) pressure waves and compression waves traveling at the speed of sound (e.g., Ref. [57]). However, similar features are consistently observed in 2D and 3D models, that do not incorporate fast compressional waves. The fearless reader is referred to Zweig [32] for a proper mathematical treatment.

15 Reducing both the damping of the partition (*ζ*) and the strength of the active force (*τ*) yields a better match to the empirical phase variation, both *in vivo* and post mortem. However, this change makes the magnitude cut-off much steeper than seen in the data. On the other hand, increasing both *ζ* and *τ* yields a better match to the shape of the transfer function above CF but produces too much phase difference between the *in vivo* and post-mortem conditions.

16 The intensity dependence of the phase evident in both the measured and predicted transfer functions is largest above CF, where the magnitude of the response is greatly attenuated relative to its peak. For this reason, the modest phase variation above CF has negligible effect on the fine structure of BM synthetic impulse responses.

17 Models in the first class often emerge from detailed considerations of cochlear anatomy and assumptions about micromechanical motions. Because errors in this realm—where there are necessarily more unknowns and fewer functional constraints—may yield unrealistic macroscopic behavior, it may be more efficient to proceed by first using macromechanical models to identify micromechanical constraints than to work the other way around.

18 This is not surprising as the ratio reflects the magnitude of the BM impedance, which reaches a minimum near CF. In particular, in the low-level linear regime, the ratio between active force and BM velocity is *iωτP*/*V*_BM_ ∝ *iω*/*Y*_BM_.

19 A second transition region, from short-wave back to long-wave behavior, occurs at frequencies above CF (or, equivalently, at locations apical to the best place). Because the region of validity of the short-wave model is limited, it is difficult to predict precisely how the wavelength varies in this region. Nevertheless, the post-mortem wavelength evidently increases enough beyond CF that it once again becomes larger than the height of the scalae. In this region beyond the peak of the traveling wave, the imaginary part of the wave number [see Fig. 7(b)] becomes large enough that neither the short- nor the long-wave approximations are especially meaningful.

20 Although the wavelengths and gain functions in the gerbil model are very similar to those in the mouse model, the net power amplification in the gerbil model is smaller than in the mouse (only ~6 dB). Note further that net power amplification in the 2D box model is not very meaningful, as in that model the BM spans the entire width of the scala, reducing the “geometric” gain.

## APPENDIX A: ALTERNATE DERIVATION OF ZWEIG’S OSCILLATOR EQUATION

This Appendix presents a simplified and rather heuristic derivation of the BM oscillator equation [Eqs. (3) and (4)] first suggested by Zweig [32]. Unlike Zweig, who obtained the result by fitting a BM admittance to measured transfer functions in the linear regime, we approach the problem by invoking the approximate intensity invariance of the phase of BM frequency responses measured in the mammalian cochlea. Whereas the magnitude of the BM frequency response undergoes large changes with level, the phase changes comparatively little (see, e.g., Figs. 4 and 5). The same approximate invariance is evident between transfer functions measured *in vivo* and post mortem.

A particular manifestation of phase-invariance is the approximate invariance of the zero crossings of BM responses to clicks [2]. Zero-crossing invariance requires that both (i) the characteristic *damped* (or ringing) frequency of the cochlear oscillator [2] and (ii) the wave-front delay of the traveling wave be independent of stimulus level—or, more precisely, independent of local BM motion and the strength of the internal force [Eq. (3)].

The characteristic frequency of the cochlear oscillators is the periodic component of the solution of the homogeneous equation associated with Eq. (3). The wave-front delay *τ*_wf_ for the model is given in Eq. (13). Applying the WKB approximation to the pressure wave in the long-wave region of a generic linear model with BM admittance *Y* (*s*) yields

(A1) $$\tau_{\text{wf}}\propto \underset{s\to 0}{\text{lim}}\sqrt{Y(s)/s}.$$

The simple assumption that the organ of Corti seen from the fluid consists, effectively, of a mass, dashpot, and spring leads immediately to Eq. (3) with *f*_act_ = 0. It is by now a classic result that this simple passive model cannot fit BM transfer functions measured at low sound levels *in vivo* [18,97]. However, as shown in Figs. 3–5, the passive model does reasonably approximate the transfer function measured post mortem. To find a function describing *f*_act_, we therefore proceed by assuming that the harmonic-oscillator model provides a good description of passive cochlear mechanics.

Without additional assumptions, in the low-level linear regime *f*_act_ can be expressed as the sum of a force *f*_BM_, depending on local BM motion (and its time derivatives), and a force *f*_p_, depending on pressure (and its derivatives). This decomposition applies whether the forces originate from complex local or nonlocal mechanisms.^21^ Hence Eq. (3) becomes

(A2) $$m\left({\dot{v}}_{\text{BM}}+2\zeta \omega_{\text{c}}v_{\text{BM}}+\omega_{\text{c}}^{2}\int v_{\text{BM}}dt\right)\;=f_{\text{ext}}+f_{\text{BM}}\left(\int v_{\text{BM}},v_{\text{BM}},{\dot{v}}_{\text{BM}},\ldots \right)+f_{\text{p}}(p,\dot{p},\ddot{p},\ldots ),$$

where *f*_ext_ = *bp* is the force (per unit length) due to external pressure. The two functions representing the active force can be expanded as

(A3) $$f_{\text{BM}}\left(\int v_{\text{BM}},v_{\text{BM}},{\dot{v}}_{\text{BM}},\ldots \right)\;=\alpha_{0}\int v_{\text{BM}}dt+\alpha_{1}v_{\text{BM}}+\alpha_{2}{\dot{v}}_{\text{BM}}+\cdots$$

and

(A4) $$f_{\text{p}}(p,\dot{p},\ddot{p},\ldots )=\beta_{0}p+\beta_{1}\dot{p}+\beta_{2}\ddot{p}+\cdots .$$

Constraint (i) above requires that the imaginary part of the eigenvalues of

(A5) $$m\left({\dot{v}}_{\text{BM}}+2\zeta \omega_{\text{c}}v_{\text{BM}}+\omega_{\text{c}}^{2}\int v_{\text{BM}}dt\right)\;=f_{\text{BM}}\left(\int v_{\text{BM}},v_{\text{BM}},{\dot{v}}_{\text{BM}},\ldots \right)$$

be the same as those of

(A6) $$m\left({\dot{v}}_{\text{BM}}+2\zeta \omega_{\text{c}}v_{\text{BM}}+\omega_{\text{c}}^{2}\int v_{\text{BM}}dt\right)=0.$$

Constraint (ii) requires that

(A7) $$\frac{m\omega_{\text{c}}^{2}-\alpha_{0}}{b+\beta_{0}}=\frac{m}{b}\omega_{\text{c}}^{2}.$$

The simplest solution that respects the constraints imposed by phase invariance is therefore given by the conditions

(A8) $$f_{\text{BM}}\left(\int v_{\text{BM}},v_{\text{BM}},v_{\dot{\text{B}}\text{M}},\ldots \right)=0$$

and

(A9) $$\beta_{0}=0.$$

The conclusion therefore is that the external force must be a function of the time derivatives of the pressure:

(A10) $$f_{\text{act}}(\dot{p},\ddot{p},\ldots )=\beta_{1}\dot{p}+\beta_{2}\ddot{p}+\cdots .$$

Hence, the simplest strategy (mathematically speaking) for finding an internal force term consistent with zero-crossing invariance is to assume it is a function of time derivatives of the local driving pressure. Empirically, the first term of the expansion [Eq. (A10)] proves sufficient to fit the data reasonably well.

## APPENDIX B: ADMITTANCES IN 1D AND 3D

Models that assume one-dimensional (1D) longitudinal fluid flow in the scalae are analogous to transmission lines [12,18,23,25,98]. Although the underlying long-wave assumption has long been known to break down near the peak of the traveling wave [11,38,39], satisfying 1D models can be derived from (or fit to) existing BM response data and the simplification is still commonly (and often implicitly) assumed to have little impact on model predictions [36,37]. However, as recent studies have pointed out [32,49], the mathematical form of the BM admittance obtained in 1D is radically different from that found in two or three dimensions. Without reiterating these arguments, we present a simple comparison between the nature of 1D and 3D models in Fig. 9. The results demonstrate that explaining measured BM velocity responses by modeling the cochlea in 1D requires that the active process appear as a narrowly tuned force.

The figure compares and contrasts the magnitude of the driving pressure and the BM admittance of the 1D active model of Ref. [18], with those of a 2D or 3D model with the identical BM-velocity frequency response. The models were obtained using the methods outlined in Ref. [49]. Recall that BM velocity is the product of the driving pressure and the BM admittance. The 1D model produces only a small peak in the driving pressure, and it therefore must include a rather sharply tuned BM admittance in order to yield the measured BM response. By contrast, in 2D or 3D models the driving pressure is more narrowly tuned, implying that the BM frequency response can be obtained with a more broadly tuned admittance.

## References

[1] Robles L and Ruggero MA, Mechanics of the Mammalian cochlea, Physiol. Rev 81, 1305 (2001). 11427697 10.1152/physrev.2001.81.3.1305 PMC3590856

[2] Shera CA, Intensity-invariance of fine time structure in basilar-membrane click responses: Implications for cochlear mechanics, J. Acoust. Soc. Am 110, 332 (2001). 11508959 10.1121/1.1378349

[3] Sisto R, Moleti A, and Altoè A, Decoupling the level dependence of the basilar membrane gain and phase in nonlinear cochlea models, J. Acoust. Soc. Am 138, EL155 (2015). 26328742 10.1121/1.4928291

[4] Johnstone BM, Genesis of the cochlear endolymphatic potential, in Current Topics in Bioenergetics (Elsevier, Amsterdam, 1967), Vol. 2, pp. 335–352.

[5] Sarpheshkar R, Lyon RF, and Mead CA, A low-power wide-dynamic-range analog vlsi cochlea, Analog Integr. Circ. Signal Process 16, 245 (1998).

[6] Robles L, Ruggero MA, and Rich NC, Two-tone distortion in the basilar membrane of the cochlea, Nature 349, 413 (1991). 1992342 10.1038/349413a0 PMC3579518

[7] Cooper NP, Harmonic distortion on the basilar membrane in the basal turn of the guinea-pig cochlea, J. Physiol 509, 277 (1998). 9547400 10.1111/j.1469-7793.1998.277bo.x PMC2230936

[8] Helmholtz HLF, Die Lehre von den Tonempfindungen als physiologische Grundlage für die Theorie der Musik (Vieweg, Braunschweig, 1863). 21717803

[9] von Békésy G and Wever EG, Experiments in Hearing (McGraw-Hill, New York, 1960), Vol. 8.

[10] Gold T, Hearing. II. The physical basis of the action of the cochlea, Proc. R. Soc. B 135, 492 (1948).

[11] Lighthill J, Energy flow in the cochlea, J. Fluid Mech 106, 149 (1981).

[12] Zweig G, Lipes R, and Pierce J, The cochlear compromise, J. Acoust. Soc. Am 59, 975 (1976). 1262596 10.1121/1.380956

[13] Kemp DT, Stimulated acoustic emissions from within the human auditory system, J. Acoust. Soc. Am 64, 1386 (1978). 744838 10.1121/1.382104

[14] Greenwood DD, A cochlear frequency-position function for several species—29 years later, J. Acoust. Soc. Am 87, 2592 (1990). 2373794 10.1121/1.399052

[15] Olson ES, Intracochlear pressure measurements related to cochlear tuning, J. Acoust. Soc. Am 110, 349 (2001). 11508960 10.1121/1.1369098

[16] Dong W and Olson ES, Detection of cochlear amplification and its activation, Biophys. J 105, 1067 (2013). 23972858 10.1016/j.bpj.2013.06.049 PMC3752116

[17] Neely S, Backward solution of a two-dimensional cochlear model, J. Acoust. Soc. Am 67, S75 (1980).

[18] Zweig G, Finding the impedance of the organ of Corti, J. Acoust. Soc. Am 89, 1229 (1991). 2030212 10.1121/1.400653

[19] de Boer E and Nuttall AL, The mechanical waveform of the basilar membrane. III. Intensity effects, J. Acoust. Soc. Am 107, 1497 (2000). 10738804 10.1121/1.428436

[20] Shera CA, Laser amplification with a twist: Traveling-wave propagation and gain functions from throughout the cochlea, J. Acoust. Soc. Am 122, 2738 (2007). 18189566 10.1121/1.2783205

[21] Kemp DT, Evidence of mechanical nonlinearity and frequency selective wave amplification in the cochlea, Arch. Otorhinolaryngol 224, 37 (1979). 485948 10.1007/BF00455222

[22] Brownell WE, Bader CR, Bertrand D, and De Ribaupierre Y, Evoked mechanical responses of isolated cochlear outer hair cells, Science 227, 194 (1985). 3966153 10.1126/science.3966153

[23] Neely ST and Kim D, A model for active elements in cochlear biomechanics, J. Acoust. Soc. Am 79, 1472 (1986). 3711446 10.1121/1.393674

[24] Mammano F and Nobili R, Biophysics of the cochlea: Linear approximation, J. Acoust. Soc. Am 93, 3320 (1993). 8326060 10.1121/1.405716

[25] Talmadge CL, Tubis A, Long GR, and Piskorski P, Modeling otoacoustic emission and hearing threshold fine structures, J. Acoust. Soc. Am 104, 1517 (1998). 9745736 10.1121/1.424364

[26] Ren T, He W, and Barr-Gillespie PG, Reverse transduction measured in the living cochlea by low-coherence heterodyne interferometry, Nat. Commun 7, 10282 (2016). 26732830 10.1038/ncomms10282 PMC4729828

[27] Ren T, He W, and Kemp D, Reticular lamina and basilar membrane vibrations in living mouse cochleae, Proc. Natl. Acad. Sci. USA 113, 9910 (2016). 27516544 10.1073/pnas.1607428113 PMC5024575

[28] Lee HY, Raphael PD, Park J, Ellerbee AK, Applegate BE, and Oghalai JS, Noninvasive *in vivo* imaging reveals differences between tectorial membrane and basilar membrane traveling waves in the mouse cochlea, Proc. Natl. Acad. Sci. USA 112, 3128 (2015). 25737536 10.1073/pnas.1500038112 PMC4364183

[29] Lee HY, Raphael PD, Xia A, Kim J, Grillet N, Applegate BE, Bowden AKE, and Oghalai JS, Two-dimensional cochlear micromechanics measured *in vivo* demonstrate radial tuning within the mouse organ of Corti, J. Neurosci 36, 8160 (2016). 27488636 10.1523/JNEUROSCI.1157-16.2016 PMC4971363

[30] Cooper NP, Vavakou A, and van der Heijden M, Vibration hotspots reveal longitudinal funneling of sound-evoked motion in the mammalian cochlea, Nat. Commun 9, 3054 (2018). 30076297 10.1038/s41467-018-05483-z PMC6076242

[31] Dewey JB, Applegate BE, and Oghalai JS, Amplification and suppression of traveling waves along the mouse organ of Corti: Evidence for spatial variation in the longitudinal coupling of outer hair cell-generated forces, J. Neurosci 39, 1805 (2019). 30651330 10.1523/JNEUROSCI.2608-18.2019 PMC6407303

[32] Zweig G, Linear cochlear mechanics J Acoust. Soc. Am 138, 1102 (2015). 10.1121/1.4922326 26328725

[33] Rhode WS, Observations of the vibration of the basilar membrane in squirrel monkeys using the Mössbauer technique, J. Acoust. Soc. Am 49, 1218 (1971). 10.1121/1.1912485 4994693

[34] Zweig G, Nonlinear cochlear mechanics J Acoust. Soc. Am 139, 2561 (2016). 10.1121/1.4941249 27250151

[35] Recio A and Rhode WS, Basilar membrane responses to broadband stimuli, J. Acoust. Soc. Am 108, 2281 (2000). 11108369 10.1121/1.1318898

[36] Neely ST and Rasetshwane DM, Modeling signal propagation in the human cochlea, J. Acoust. Soc. Am 142, 2155 (2017). 29092611 10.1121/1.5007719 PMC6578578

[37] Sisto R, Shera CA, Altoè A, and Moleti A, Constraints imposed by zero-crossing invariance on cochlear models with two mechanical degrees of freedom, J. Acoust. Soc. Am 146, 1685 (2019). 31590512 10.1121/1.5126514 PMC6756920

[38] Siebert WM, Ranke revisited—A simple short-wave cochlear model, J. Acoust. Soc. Am 56, 594 (1974). 4415469 10.1121/1.1903296

[39] Steele CR and Taber LA, Comparison of WKB calculations and experimental results for three-dimensional cochlear models, J. Acoust. Soc. Am 65, 1007 (1979). 447914 10.1121/1.382570

[40] Shera CA and Charaziak KK, Cochlear frequency tuning and otoacoustic emissions, Cold Spr. Harb. Persp. Med 9, a033498 (2019). 10.1101/cshperspect.a033498 PMC6360871 30037987

[41] Shera CA and Zweig G, A symmetry suppresses the cochlear catastrophe, J. Acoust. Soc. Am 89, 1276 (1991). 2030215 10.1121/1.400650

[42] Ramamoorthy S, Zha D, Chen F, Jacques SL, Wang R, Choudhury N, Nuttall AL, and Fridberger A, Filtering of acoustic signals within the hearing organ, J. Neurosci 34, 9051 (2014). 24990925 10.1523/JNEUROSCI.0722-14.2014 PMC4078082

[43] Lynch TJ, Nedzelnitsky V, and Peake WT, Input impedance of the cochlea in cat, J. Acoust. Soc. Am 72, 108 (1982). 7108034 10.1121/1.387995

[44] Burda H, Ballast L, and Bruns V, Cochlea in old world mice and rats (Muridae), J. Morphol 198, 269 (1988). 3221404 10.1002/jmor.1051980303

[45] Santi PA, Rapson I, and Voie A, Development of the mouse cochlea database (MCD), Hear. Res 243, 11 (2008). 18603386 10.1016/j.heares.2008.04.014 PMC2628570

[46] Müller M, von Hünerbein K, Hoidis S, and Smolders JW, A physiological place–frequency map of the cochlea in the CBA/J mouse, Hear. Res 202, 63 (2005). 15811700 10.1016/j.heares.2004.08.011

[47] Neely ST, Mathematical modeling of cochlear mechanics, J. Acoust. Soc. Am 78, 345 (1985). 4031241 10.1121/1.392497

[48] van der Heijden M, Frequency selectivity without resonance in a fluid waveguide, Proc. Natl. Acad. Sci. USA 111, 14548 (2014). 25237137 10.1073/pnas.1412412111 PMC4209998

[49] Shera CA, Tubis A, and Talmadge CL, Coherent reflection in a two-dimensional cochlea: Short-wave versus long-wave scattering in the generation of reflection-source otoacoustic emissions, J. Acoust. Soc. Am 118, 287 (2005). 16119350 10.1121/1.1895025

[50] Krylov VV, Acoustic black holes: Recent developments in the theory and applications, IEEE Trans. Ultrason. Ferroelectr. Freq. Control 61, 1296 (2014). 25073137 10.1109/TUFFC.2014.3036

[51] Neely ST, Finite difference solution of a two-dimensional mathematical model of the cochlea, J. Acoust. Soc. Am 69, 1386 (1981). 7240568 10.1121/1.385820

[52] Plassmann W, Peetz W, and Schmidt M, The cochlea in gerbilline rodents, Brain Behav. Evol 30, 82 (1987). 3620898 10.1159/000118639

[53] Kanis LJ and de Boer E, Self-suppression in a locally active nonlinear model of the cochlea: A quasilinear approach, J. Acoust. Soc. Am 94, 3199 (1993). 8300954 10.1121/1.407225

[54] Smith DR, Singular-Perturbation Theory: an Introduction with Applications (Cambridge University Press, Cambridge, UK, 1985).

[55] Dong W and Olson ES, Middle ear forward and reverse transmission in gerbil, J. Neurophysiol 95, 2951 (2006). 16481455 10.1152/jn.01214.2005

[56] Dong W, Varavva P, and Olson ES, Sound transmission along the ossicular chain in common wild-type laboratory mice, Hear. Res 301, 27 (2013). 23183032 10.1016/j.heares.2012.11.015 PMC3669248

[57] Recio-Spinoso A and Rhode WS, Fast waves at the base of the cochlea, PLoS ONE 10, e0129556 (2015). 26062000 10.1371/journal.pone.0129556 PMC4465671

[58] Shera CA and Cooper NP, Basilar-membrane interference patterns from multiple internal reflection of cochlear traveling waves, J. Acoust. Soc. Am 133, 2224 (2013). 23556591 10.1121/1.4792129 PMC4109360

[59] de Boer E, Connecting frequency selectivity and nonlinearity for models of the cochlea, Aud. Neurosci 3, 377 (1997).

[60] Steele CR, Boutet de Monvel J, and Puria S, A multiscale model of the organ of Corti, J. Mech. Mater. Struct 4, 755 (2009). 20485573 10.2140/jomms.2009.4.755 PMC2871772

[61] Meaud J and Grosh K, The effect of tectorial membrane and basilar membrane longitudinal coupling in cochlear mechanics, J. Acoust. Soc. Am 127, 1411 (2010). 20329841 10.1121/1.3290995 PMC2856508

[62] Kapuria S, Steele CR, and Puria S, Unraveling the mystery of hearing in gerbil and other rodents with an arch-beam model of the basilar membrane, Sci. Rep 7, 228 (2017). 28331175 10.1038/s41598-017-00114-x PMC5427805

[63] Bowling T, Lemons C, and Meaud J, Reducing tectorial membrane viscoelasticity enhances spontaneous otoacoustic emissions and compromises the detection of low level sound, Sci. Rep 9, 7494 (2019). 31097743 10.1038/s41598-019-43970-5 PMC6522542

[64] Zhou W and Nam J-H, Probing hair cell’s mechanotransduction using two-tone suppression measurements, Sci. Rep 9, 4626 (2019). 30874606 10.1038/s41598-019-41112-5 PMC6420497

[65] Sasmal A and Grosh K, Unified cochlear model for low- and high-frequency mammalian hearing, Proc. Natl. Acad. Sci. USA 116, 13983 (2019). 31221750 10.1073/pnas.1900695116 PMC6628805

[66] de Boer E and Nuttall AL, The ‘inverse problem’ solved for a three-dimensional model of the cochlea. III. Brushing-up the solution method, J. Acoust. Soc. Am 105, 3410 (1999). 10380664 10.1121/1.424669

[67] Lamb JS and Chadwick RS, Dual Traveling Waves in An Inner Ear Model with Two Degrees of Freedom, Phys. Rev. Lett 107, 088101 (2011). 21929207 10.1103/PhysRevLett.107.088101 PMC3508461

[68] Duifhuis H, Hoogstraten H, Van Netten S, Diependaal R, and Bialek W, Modelling the cochlear partition with coupled van der Pol oscillators, in Peripheral Auditory Mechanisms (Springer-Verlag, New York, 1986), pp. 290–297.

[69] Camalet S, Duke T, Jülicher F, and Prost J, Auditory sensitivity provided by self-tuned critical oscillations of hair cells, Proc. Natl. Acad. Sci. USA 97, 3183 (2000). 10737791 10.1073/pnas.97.7.3183 PMC16213

[70] Duke T and Jülicher F, Active Traveling Wave in the Cochlea, Phys. Rev. Lett 90, 158101 (2003). 12732074 10.1103/PhysRevLett.90.158101

[71] Zweig G and Shera CA, The origin of periodicity in the spectrum of evoked otoacoustic emissions, J. Acoust. Soc. Am 98, 2018 (1995). 7593924 10.1121/1.413320

[72] Shera CA, Guinan JJ, and Oxenham AJ, Revised estimates of human cochlear tuning from otoacoustic and behavioral measurements, Proc. Natl. Acad. Sci. USA 99, 3318 (2002). 11867706 10.1073/pnas.032675099 PMC122516

[73] Moleti A and Sisto R, Comparison between otoacoustic and auditory brainstem response latencies supports slow backward propagation of otoacoustic emissions, J. Acoust. Soc. Am 123, 1495 (2008). 18345838 10.1121/1.2836781

[74] Verhulst S, Bharadwaj H, Mehraei G, Shera C, and Shinn-Cunningham B, Functional modeling of the human auditory brainstem response to broadband stimulation, J. Acoust. Soc. Am 138, 1637 (2015). 26428802 10.1121/1.4928305 PMC4592442

[75] Verhulst S, Altoè A, and Vasilkov V, Computational modeling of the human auditory periphery: Auditory-nerve responses, evoked potentials and hearing loss, Hear. Res 360, 55 (2018). 29472062 10.1016/j.heares.2017.12.018

[76] Geisler CD and Sang C, A cochlear model using feed-forward outer-hair-cell forces, Hear. Res 86, 132 (1995). 8567410 10.1016/0378-5955(95)00064-b

[77] Chen F, Zha D, Fridberger A, Zheng J, Choudhury N, Jacques SL, Wang RK, Shi X, and Nuttall AL, A differentially amplified motion in the ear for near-threshold sound detection, Nat. Neurosci 14, 770 (2011). 21602821 10.1038/nn.2827 PMC3225052

[78] He W, Kemp D, and Ren T, Timing of the reticular lamina and basilar membrane vibration in living gerbil cochleae, eLife 7, e37625 (2018). 30183615 10.7554/eLife.37625 PMC6125122

[79] Cody A, Acoustic lesions in the mammalian cochlea: Implications for the spatial distribution of the ‘active process’, Hear. Res 62, 166 (1992). 1429258 10.1016/0378-5955(92)90182-m

[80] Fisher JA, Nin F, Reichenbach T, Uthaiah RC, and Hudspeth A, The spatial pattern of cochlear amplification, Neuron 76, 989 (2012). 23217746 10.1016/j.neuron.2012.09.031 PMC3721062

[81] Pang XD and Guinan JJ, Growth rate of simultaneous masking in cat auditory-nerve fibers: Relationship to the growth of basilar-membrane motion and the origin of two-tone suppression, J. Acoust. Soc. Am 102, 3564 (1997). 9407650 10.1121/1.420147

[82] LeMasurier M and Gillespie PG, Hair-cell mechanotransduction and cochlear amplification, Neuron 48, 403 (2005). 16269359 10.1016/j.neuron.2005.10.017

[83] Dallos P, Cochlear amplification, outer hair cells and prestin, Curr. Opin. Neurobiol 18, 370 (2008). 18809494 10.1016/j.conb.2008.08.016 PMC2630119

[84] Ashmore J, Avan P, Brownell W, Dallos P, Dierkes K, Fettiplace R, Grosh K, Hackney C, Hudspeth A, Jülicher F , The remarkable cochlear amplifier, Hear. Res 266, 1 (2010). 20541061 10.1016/j.heares.2010.05.001 PMC6366996

[85] Hudspeth A, Jülicher F, and Martin P, A critique of the critical cochlea: Hopf—a bifurcation—is better than none, J. Neurophysiol 104, 1219 (2010). 20538769 10.1152/jn.00437.2010 PMC2944685

[86] Nobili R and Mammano F, Biophysics of the cochlea II: Stationary nonlinear phenomenology, J. Acoust. Soc. Am 99, 2244 (1996). 8730071 10.1121/1.415412

[87] Scherer MP and Gummer AW, Impedance analysis of the organ of Corti with magnetically actuated probes, Biophys. J 87, 1378 (2004). 15298940 10.1529/biophysj.103.037184 PMC1304476

[88] Scherer MP and Gummer AW, Vibration pattern of the organ of Corti up to 50 kHz: Evidence for resonant electromechanical force, Proc. Natl. Acad. Sci. USA 101, 17652 (2004). 15591348 10.1073/pnas.0408232101 PMC535427

[89] Cooper N and Dong W, Baseline position shifts and mechanical compression in the apical turns of the cochlea, in Biophysics of the Cochlea: From Molecules to Models (World Scientific, Singapore, 2003), pp. 261–270.

[90] Cormack J, Liu Y, Nam J-H, and Gracewski SM, Two-compartment passive frequency domain cochlea model allowing independent fluid coupling to the tectorial and basilar membranes, J. Acoust. Soc. Am 137, 1117 (2015). 25786927 10.1121/1.4908214 PMC5848829

[91] Elliott SJ, Ni G, and Sun L, Fitting pole-zero micromechanical models to cochlear response measurements, J. Acoust. Soc. Am 142, 666 (2017). 28863604 10.1121/1.4996128

[92] Klapuri A, Multipitch analysis of polyphonic music and speech signals using an auditory model, IEEE Trans. Audio, Speech, Lang. Proc 16, 255 (2008).

[93] Lyon RF, Cascades of two-pole–two-zero asymmetric resonators are good models of peripheral auditory function, J. Acoust. Soc. Am 130, 3893 (2011). 22225045 10.1121/1.3658470

[94] Takanen M, Santala O, and Pulkki V, Binaural assessment of parametrically coded spatial audio signals, in The Technology of Binaural Listening (Springer, Berlin, 2013), pp. 333–358.

[95] Jürgens T, Clark NR, Lecluyse W, and Meddis R, Exploration of a physiologically-inspired hearing-aid algorithm using a computer model mimicking impaired hearing, Int. J. Audiol 55, 346 (2016). 26918797 10.3109/14992027.2015.1135352

[96] Baby D and Verhulst S, Biophysically-inspired features improve the generalizability of neural network-based speech enhancement systems, in Proceedings of the Interspeech (International Speech Communication Association, Hyderadab, India, 2018), pp. 3264–3268.

[97] de Boer E, On active and passive cochlear models—Toward a generalized analysis, J. Acoust. Soc. Am 73, 574 (1983). 6841796 10.1121/1.389003

[98] Zwislocki JJ, Über die mechanische Klanganalyse des Ohrs, Experientia 2, 415 (1946). 20291780 10.1007/BF02154224

